# The importance of time perspective in media multitasking behavior

**DOI:** 10.3389/fpsyg.2025.1654790

**Published:** 2025-10-31

**Authors:** Alena A. Rogojina, Justin Kantner, Douglas A. Gentile

**Affiliations:** ^1^Department of Psychology, Iowa State University of Science and Technology, Ames, IA, United States; ^2^Department of Psychology, California State University Northridge, Northridge, CA, United States

**Keywords:** media multitasking, individual differences, immediate gratification, self-regulation, time perspective

## Abstract

**Introduction:**

Media multitasking (using several forms of media at once or using media during a non-media activity) occurs frequently in daily life, though some multitask more than others. This study investigated how individual differences in tendency toward immediate gratification, conceptualized using dual-process and dual-motive models of self-control, are associated with frequency of media multitasking behavior.

**Methods:**

This report extends existing knowledge and offers a comprehensive view by combining self-report survey measures with objective behavioral tasks in two U. S. student samples (Study 1 from a Hispanic-Serving Institution, *N* = 487; and Study 2 from a Midwestern research university, *N* = 381). Participants completed self-report measures of media multitasking frequency, effortful control, mindfulness, and time perspective (future versus immediate-goal focus). They also answered retrospective time estimation questions and completed a Time Production (in Study 1 only) and Stop Signal task using E-Prime Go. Individual multitasking scores, media combinations, and in-study multitasking were also examined, and in Study 2 participants also completed a delay discounting measure.

**Results:**

Components of cognitive control had significant negative associations with media multitasking behavior. The strongest positive associations were with having a present-focused time perspective and favoring immediate over distal rewards. Issues with time estimation played a role as well.

**Discussion:**

Overall, our findings suggest that a *preference* for immediate reward might outweigh cognitive control *ability* when predicting media multitasking behavior.

## Introduction

Media multitasking (i.e., using two or more forms of media at once or engaging with media while performing another activity) is a phenomenon that permeates the daily lives of many emerging adults (Beuckels et al., 2021; Ophir et al., 2009). It might manifest as checking notifications while completing homework or watching a show while cooking. In some situations, like using one’s phone while driving, it can have dire consequences. Some people multitask more than others. Increased rates of media multitasking have been found to be associated with attentional difficulties, socioemotional problems, and decreased academic performance (van der Schuur et al., 2015 for a review). What might account for some of these differences in media multitasking frequency? Prior research has pointed to the role of demographics and personality differences (Duff et al., 2014) and motivations for use (Wang and Tchernev, 2012). However, there has been limited investigation into the sense of immediate gratification media multitasking can provide, and individuals’ predisposition toward attaining it.

The current media landscape offers an opportunity for constant stimulation. By pausing an activity to check an incoming notification or switching attention frequently between two or more streams of information, users can receive an instantaneous feeling of reward. In line with this, those who tend to multitask frequently have been found to do worse on delay discounting tasks (Schutten et al., 2017), tending to choose small immediate monetary rewards over larger ones at a delayed time, and a preference for immediate gratification has been found to underlie off-task media use in the classroom (Hayashi and Blessington, 2018). In an fMRI study, Lopez et al. (2020) found media multitasking frequency to be associated with greater activation in the reward centers of the brain compared to the self-regulation centers. One of the goals of this report is to investigate media multitasking behavior as an outcome of self-control tendencies, using Hofmann et al. (2009) dual-process model and a multimethod approach. We plan to investigate some of the mechanisms underlying greater media multitasking behavior.

### Immediate gratification and self-control

Self-control can be conceptualized as a dual-process model (Hofmann et al., 2009). An automatic and impulsive System 1 competes with a reflective and deliberate System 2. System 1 behavior favors immediate gratification, or the attainment of short-term hedonic goals over long-term instrumental ones. Hofmann et al. (2009) recommended measuring individual differences in both the impulsive and reflective systems when investigating self-control. The impulsive system can be captured with behavioral measures and performance assays that examine impulsivity in responding, and the reflective system with self- and other-reports of self-regulatory behavior. Statistically, the two are related but contribute unique variance to explaining behavior. Though everyone indulges System 1 impulsivity occasionally, a preference for this behavior over System II regulation has been associated with self-harm, substance use, and behavioral addictions (Ioannidis et al., 2019; Kozak et al., 2019; McHugh et al., 2019). This dual-process conceptualization of self-control is used as a framework to guide our hypotheses.

### Systems 1 and 2 self-control and media multitasking

Cognitive control is conceptualized as both a domain-general ability and one that can be broken down into three subcomponents: updating (monitoring working memory), shifting (switching between tasks or goals), and inhibition (controlling responses rather than acting on the basis of automatic processing; Miyake et al., 2000). Inhibition underlies successful control of updating and shifting, and is key in subverting the pursuit of immediate gratification (Wegmann et al., 2020). It is often measured using performance assays like the Go/No-Go and Stop Signal Tasks, requiring participants to press the correct buttons when needed and withhold responding during a “stop” indicator (Gratton et al., 2018). It can be viewed as an analog of System 1 impulsivity in the dual-process model.

One’s trait-level tendency toward employing cognitive control to regulate one’s behavior, analogous to System 2 self-control, can be referred to as effortful control (Nigg, 2017). Successful effortful control underlies impulse control, planning, attentional abilities, and emotional regulation (Meehan et al., 2013). Effortful control encompasses inhibitory control and attentional control, similar to the inhibition and shifting subcomponents of cognitive control, as well as activation control (i.e., purposefully beginning to engage in necessary tasks; Evans and Rothbart, 2007). Inhibitory control is of particular interest, as it mirrors the System 1 inhibitory control measure discussed above. An incoming phone notification might inspire a prepotent response of checking the phone immediately, for example, especially if this behavior pattern is common. Those with greater inhibitory control would be better able to stop themselves. Interestingly, System 1 cognitive control has been found to moderate the relation between self-reported self-regulation and health and wellbeing outcomes (Hakun and Findeison, 2020).

Ophir et al. (2009) found that heavy media multitaskers have lowered cognitive control capabilities compared to light media multitaskers, which manifests as difficulty filtering distractions (i.e., inhibition) and switching between tasks (i.e., shifting). However, recent meta-analyses on the relation between cognitive control and media multitasking have found mixed results (Kong et al., 2023; Parry and Le Roux, 2021; Wiradhany and Koerts, 2021). Although self-reported cognitive control issues showed small to medium associations with media multitasking, the meta-effect of most performance-based assays was small and non-significant. This may reflect a difference between typical and optimal performance: Media multitasking may not be associated with having difficulty performing a specific cognitive task for a finite amount of time, but instead with longer-term everyday lapses in control. For example, not being able to successfully regulate one’s behavior or pay attention for extended periods of time in an uncontrolled environment (Wiradhany and Koerts, 2021).

We hypothesize that greater effortful control in general (H1) and inhibitory control in particular (H2a: measured using a performance-based assay; H2b: measured using self-report) will be associated with less media multitasking behavior. Activation control has not previously been examined in the context of media multitasking, so we also intend to determine whether it is associated with the behavior (RQ1).

### Mindfulness and media multitasking

Underlying cognitive control abilities have frequently been studied alongside media multitasking behaviors. However, a related trait-level construct emblematic of System 2 self-control that may be worth investigating is mindfulness. Successful mindfulness requires the practitioner to have adequate cognitive control in order to be able to maintain present-centered awareness and not be carried away by thoughts and distractions. This is relevant to media multitasking, as those who media multitask more frequently may, ironically, be less able to filter distractions and focus on one thing at a time (Ophir et al., 2009). Lower mindfulness is associated with more problematic media use, and greater mindfulness has been found to moderate the relation between media use and negative mental health outcomes (Meynadier et al., 2024; Stratton et al., 2022). Some mind wandering studies have examined the relation between media multitasking and the acting with awareness facet of the construct (i.e., focusing one’s attention deliberately; Ralph et al., 2014), finding negative associations. No studies, however, have examined the associations between mindfulness as a whole or its other facets: observing of internal and external experiences, describing or labeling these experiences, acting with awareness, nonjudging of thoughts and emotions, and nonreacting to thoughts and emotions. We expect greater trait-level mindfulness to be associated with lower levels of media multitasking (H3) and will examine the relations between media multitasking and the individual mindfulness facets (RQ2).

### Time-related variables

An alternative to the dual-process framework proposed by Hofmann et al. (2009) is the dual-*motive* perspective of self-control (Fujita, 2011). In any situation that requires self-control, an individual must choose between pursuing either concrete short-term or more abstract long-term goals. Tendencies toward one or the other can be conceptualized as individual differences in time perspective: a preference for either dwelling on the past, living in the moment/focusing on short-term goals, or planning for the future (Zimbardo and Boyd, 1999). Another goal of this report is to examine Fujita (2011) model of self-control alongside Hofmann et al. (2009) to see which better explains media multitasking behavioral tendencies.

Time perspective, or preference, is often examined as a personality trait (Zimbardo and Boyd, 1999). Having a future-focused time perspective, compared to one focused on either the past or on short-term rewards, is associated with greater achievement (e.g., of school and work-related goals) and wellbeing, as well as fewer risky behaviors (Kooij et al., 2018). Having a present-oriented time perspective has been found to be associated with choosing short-term over long-term rewards on the Delay Discounting Task (Acuff et al., 2017) and Money Choice Questionnaire (Daugherty and Brase, 2010) in college student samples.

A study that investigated media multitasking in the university classroom found that frequency of multitasking was negatively associated with having a future time perspective, or being future goal oriented (Labăr and Ţepordei, 2019). Problematic phone use mediated this relation. To date, this has been the only study investigating time perspective in media multitasking. Another has found problematic or excessive media use in general to be associated with being short-term goal focused (Settanni et al., 2018). The present study will be the first to examine media multitasking tendencies, rather than a specific multitasking situation, alongside individual differences in time perspective. We expect that having an immediate-focused time perspective will be associated with more media multitasking behavior (H4).

Both System 1 and System 2 measures of self-control interact with measures of time perspective. In one study, self-control abilities mediated the relation between time perspective and both procrastination and internet addiction (Kim et al., 2017). Self-control also sometimes moderates the relation between time perspective and achievement outcomes (Barber et al., 2009), such that having lower self-control makes the associations stronger.

In addition to time perspective, which is inherently tied to explicit value judgments and goal-setting, there is the concept of the internal clock (Church, 1984). This can be viewed as another performance-based measure of System 1 impulsivity. Both over- and underestimation of elapsed time durations is associated with individual differences in impulsivity, attention regulation, and working memory (Block, 2014; Dougherty et al., 2005). A less balanced time perspective is also associated with less accuracy in time estimation (Witowska et al., 2020). The same study found that inhibitory control moderated the relation between unbalanced time perspective and time estimation.

We will investigate whether this measure of impulsivity is associated with more or less media multitasking behavior. While no studies to date have investigated the relation between individual differences in media multitasking and time passage estimation ability, an experimental multitasking paradigm that required participants to watch a video advertisement while monitoring a second stimulus in another window found that participants underestimated the passage of time (Chinchanachokchai et al., 2015). In another experimental study, participants who rapidly switched their attention between a high- (e.g., a sitcom) and a low-entertainment stimulus (e.g., an academic article) more frequently stated that time “flew by,” as opposed to “dragging on” in the single-stream conditions (Xu and David, 2018). These findings point to real-time media multitasking being associated with time passage underestimation (H5). We will also examine time over- and underestimation tendency using a performance-based assay (RQ3). This will be the first study to do so in the context of media multitasking.

### The current study

This study will use Hofmann et al. (2009) dual-process model alongside Fujita (2011) dual-motive model of self-control to investigate immediate gratification in media multitasking behavior. Prior studies have examined cognitive control, mindfulness, time perspective, and time estimation in the context of media multitasking to varying degrees, though not together; thus, the relative contributions of these attributes to media multitasking behaviors could not be established. The current study will add to the existing media multitasking and self-control literatures in a holistic way by using both self-report and behavioral measures of the variables of interest. We will also investigate which of these constructs explains the greatest amount of variance in media multitasking behavior. Finally, we will test these hypotheses in two geographically and culturally distinct U. S. student samples. There have been heterogeneous findings on the relation between media multitasking and cognitive control, and limited investigation into the other constructs, so using two large and well-powered samples should prove useful to the literature.

## Study 1

### Materials and methods

#### Participants

A total of 536 participants took part in the study in early 2022. Participants were recruited through the university’s psychology research subject pool and were compensated with course credit. Demographics-based barriers to participation were minor, in that participants were required to be between 18 and 29 years old (i.e., emerging adults; Arnett et al., 2014) and have normal or corrected-to-normal vision. The data were originally collected as part of a master’s thesis and meant to be analyzed using structural equation modeling methods. Best practices dictate a minimum of 10 cases of observation per measured variable (Tabachnick et al., 2007). However, a larger sample size grants more statistical power. The intent was to recruit a minimum of 250 participants, but to leave the study open for sign-ups and leave data untouched until the subject pool closed at the end of the semester. To assess resulting power, we conducted sensitivity analyses for all hypotheses in Study 1 (see Supplementary material).

After removing incomplete responses (i.e., those who completed less than 33% of the survey; *N* = 19), those who failed at least two out of three attention checks (*N =* 26), and those over 29 years of age (*N* = 4), we were left with 487 participants in the final analysis. Sample descriptive statistics can be found in Table 1.

**Table 1:** Participant characteristics in studies 1 and 2.

Demographics	Study 1 (*N* = 487)		Study 2 (*N* = 381)	
	*N*	%	*N*	%
Gender	485	99.69^a^	381	100.00
Woman	318	65.30	229	60.10
Man	156	32.03	149	39.11
Another gender identity	11	2.26	3	0.79
Race/Ethnicity	487	100.00	381	100.00
Hispanic/Latine	287	58.93	26	6.82
White/Caucasian Euro	50	10.27	233	61.15
Asian/Asian American	49	10.06	23	6.044
White/Caucasian Middle Eastern	36	7.9	60	15.75
Black/African American	26	5.34	26	6.82
Multiethnic or another race/ethnicity	39	8.01	13	3.41
Classification	487	100.00	381	100.00
Freshman	309	63.45	220	57.74
Sophomore	92	18.89	91	23.88
Junior	53	10.88	49	12.86
Senior	32	6.57	20	5.25
Other			1	0.26

	*N*	%	*M*	*SD*	Range
Age					
Study 1	487	100.00	19.22	1.71	18–29
Study 2	381	100.00	19.23	1.19	18–26
Financial Stress					
Study 1	432	88.70	44.73	28.53	0–100
Study 2	380	99.74	40.16	28.79	0–100

^a^Two participants did not provide gender data.

Approximately one third (*N* = 184) of participants did not complete the E-Prime portion of the study because their computers did not meet operating system requirements, and so did not provide data for the Stop Signal and Time Production Tasks. Those who did and those who did not complete the E-Prime portion did not differ significantly on media multitasking tendency [*t*(479) = 1.259, *p* = 0.209] or level of multitasking while completing the survey [*t*(484) = 0.928, *p* = 0.354], so were grouped together for some analyses.

#### Procedure

Participants completed an informed consent and a series of questionnaires on Qualtrics. Participants were then automatically redirected to the E-Prime Go website (Psychology Software Tools, Inc., 2020) and instructed to download and run the experiment file, which contained the two tasks described below in *Behavioral Measures*. This procedure was first pilot tested (*N* = 31) for feasibility. Due to COVID-19 related restrictions, participants took part in the study on their own personal devices instead of in a research lab environment. The survey took approximately 34 min and the E-Prime portion was designed to take 10 min, though the actual time was not recorded due to a technical error.

#### Measures

##### Survey measures

The survey contained validated scales, four attention check questions, two questions about real-time multitasking during the survey and E-Prime task portions of the study (i.e., “in-study multitasking”), and two retrospective time estimation questions (described further in *Behavioral Measures*). The participants provided demographics data through a separate mass testing survey. Higher scores on all scales indicated a higher level of the construct. Factor analyses for all scale variables other than the Media Multitasking Index can be found in Supplementary material.

###### Media Multitasking Index

The Media Multitasking Index (MMI) (Ophir et al., 2009) is an often-used self-report measure that requires participants to estimate the number of hours per average day they spend engaging in 10 media and non-media activities (i.e., the primary activity), then to estimate the percentage of time they spend co-engaging with a secondary activity from the same list. An updated version of the MMI that was more reflective of current technological trends and relevant to university students was used (Ralph and Smilek, 2017). The 10 activities were: (1) talking face-to-face with a person, (2) using print media, (3) texting, instant messaging, or emailing, (4) using social sites, (5) using non-social text-oriented sites, (6) talking on the phone or video chatting, (7) listening to audio content, (8) watching video content, (9) playing video games or online games, and (10) doing homework/studying/writing papers. Participants were asked to state if they estimated the times or if they consulted with screen time monitoring programs (e.g., Screen Time, Digital Wellbeing) they had installed on their devices.

The MMI was scored following Ophir et al. (2009) original formula, where *m_i_* is the number of activities in which a participant typically engaged alongside the primary activity *i*, *h_i_* is the number of hours per day spent on primary activity *i*, and *h_total_* is the sum of hours spent on all primary media. An example of this calculation can be found in Supplementary material. The current study treated the MMI score as a continuous variable.


$$\mathrm{MMI}=\sum_{i=1}^{10}\frac{m_{i\times h_{i}}}{h_{\text{total}}}$$

A concern with using only one aggregate measure of the MMI is that nuanced information about the extent and the type of multitasking is lost (Parry and Fisher, 2025). As such, we conducted additional analyses with individual activity scores (i.e., each activity as a main and a background activity) and multitasking combinations. Background activity scores were calculated by finding the average percentage of time the activity was used in the background across all primary activities.

###### Adult Temperament Questionnaire-Effortful Control (α = 0.74)

The effortful control subscale of the Adult Temperament Questionnaire-Short Form (ATQ-SF) (Evans and Rothbart, 2007) was used to assess trait effortful control. The subscale contains 19 questions rated on a 7-point Likert scale (“extremely untrue” to “extremely true”) and can be further broken down into three facets: activation control (e.g., “I can make myself work on a difficult task even when I do not feel like trying.”; 7 questions; *α* = 0.61), attentional control (e.g., “When I am trying to focus my attention, I am easily distracted (reverse scored); 5 questions; α = 0.65), and inhibitory control (e.g., “It is easy for me to inhibit fun behavior that would be inappropriate.”; 7 questions; α = 0.42).

###### Five Facet Mindfulness Questionnaire (α = 0.86)

The Five Facet Mindfulness Questionnaire (FFMQ) (Baer et al., 2006) was used to assess trait mindfulness. The FFMQ is composed of 39 questions on a five-point scale (“never or very rarely true” to “very often or always true”). The scale has a five-factor structure, with each factor corresponding to a facet of mindfulness: observing (e.g., “When I’m walking, I deliberately notice the sensations of my body moving.”; 8 questions; *α* = 0.76), describing (e.g., “I’m good at finding words to describe my feelings.”; 8 questions; α = 0.85), acting with awareness (e.g.,” When I do things, my mind wanders off and I’m easily distracted (reverse scored); 8 questions; α = 0.85), nonjudging (e.g.,” I criticize myself for having irrational or inappropriate emotions (reverse scored); 8 questions; α = 0.87), and nonreactivity (e.g.,“I perceive my feelings and emotions without having to react to them.”; 7 questions; α = 0.72).

###### Considerations of Future Consequences Scale

Orientation toward the future and immediate present time perspectives was assessed using the updated Considerations of Future Consequences Scale (CFC) (Joireman et al., 2012). The CFC consists of 14 questions rated on a 7-point Likert scale (1 “not at all like me” to 7 “very much like me”) that support either a concern for future (e.g., “I consider how things might be in the future, and try to influence those things with my day-to-day behavior”) or more immediate (e.g., “Since my day-to-day work has specific outcomes, it is more important to me than behavior that has distant outcomes”) consequences. The updated 14-question scale was used in this study because it has clear support for a two-factor solution. The internal consistency was acceptable (Future: α = 0.78, Immediate: α = 0.81).

##### Behavioral measures

Two performance-based assays were created in E-Prime 3.0 (Psychology Software Tools, Inc., 2016) and delivered to participants’ computers via E-Prime Go (Psychology Software Tools, Inc., 2020). We also asked participants to report on their media multitasking behavior during the two portions of the study (i.e., in-study multitasking, yes/no and description of the activity) and to estimate how much time had elapsed at the midpoint and end of the survey. Time estimation accuracy was calculated by dividing the participants’ estimate by the objective amount of time that had passed. Values above 1 indicated overestimation of elapsed time, while values below 1 indicated underestimation.

###### Stop Signal Task

**Figure 1:**
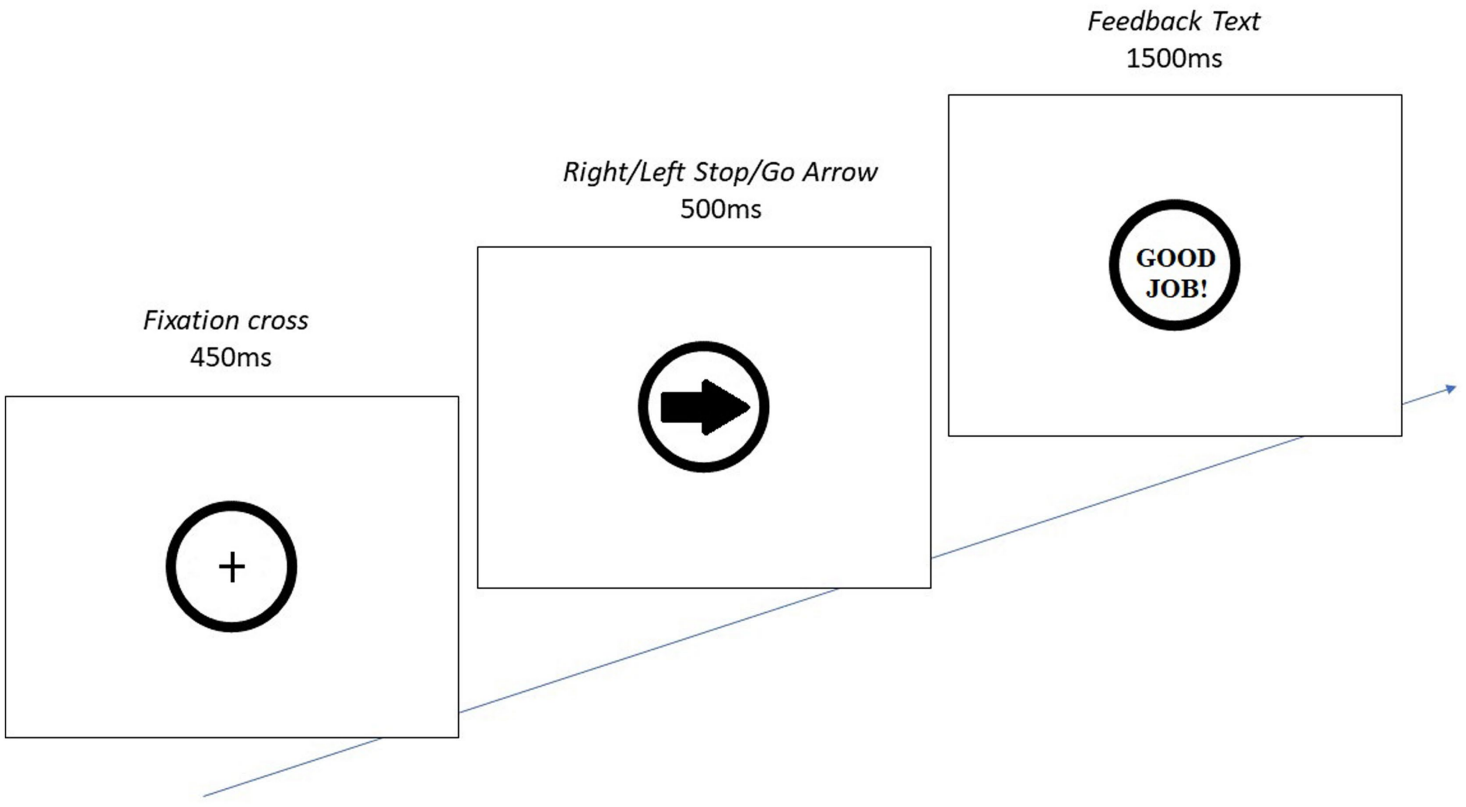
*The stimulus presented is either a right-facing go-arrow (shown), left-facing go-arrow, or right- or left-facing stop-arrow (red ring around arrow instead of black). Participants must press the right [left] arrow on the keyboard when a right [left] arrow is shown, and press nothing when a stop-arrow is shown. The fixation cross indicates where the arrow will appear. **The feedback text either reads “Good Job!” (shown) if participant correctly did not respond on a stop-arrow or pressed the correct key on either go-arrow, “Incorrect!” if participant pressed incorrect key on go-arrow (e.g., right key for left arrow), “Too Slow!” if participant did not respond in 500 ms or less on a go-arrow, or “Do not Respond!” if participant responded on a stop-arrow.

A Stop Signal Task (SST) available on the online Psychology Software Tools experiment repository was used to measure response inhibition (Logan and Cowan, 1984; Psychology Software Tools, Inc., 2022). Participants first completed a training phase, after which they were informed of their performance. There were 80 trials in the experimental phase, with all stimuli presented in a random order: 20 each of left and right *go* arrows and 20 each of left and right *stop* arrows. The trials paused and participants had an opportunity for a break at the midway point. The procedure for the experimental phase is illustrated in Figure 1.

Data collected from this task included *go* arrow reaction time (ms), omission errors (i.e., both failure to respond and failure to respond correctly on *go* trials), and commission errors (i.e., responding on *stop* trials). Fewer errors indicated greater inhibitory control.

###### Time Production Task

**Figure 2:**
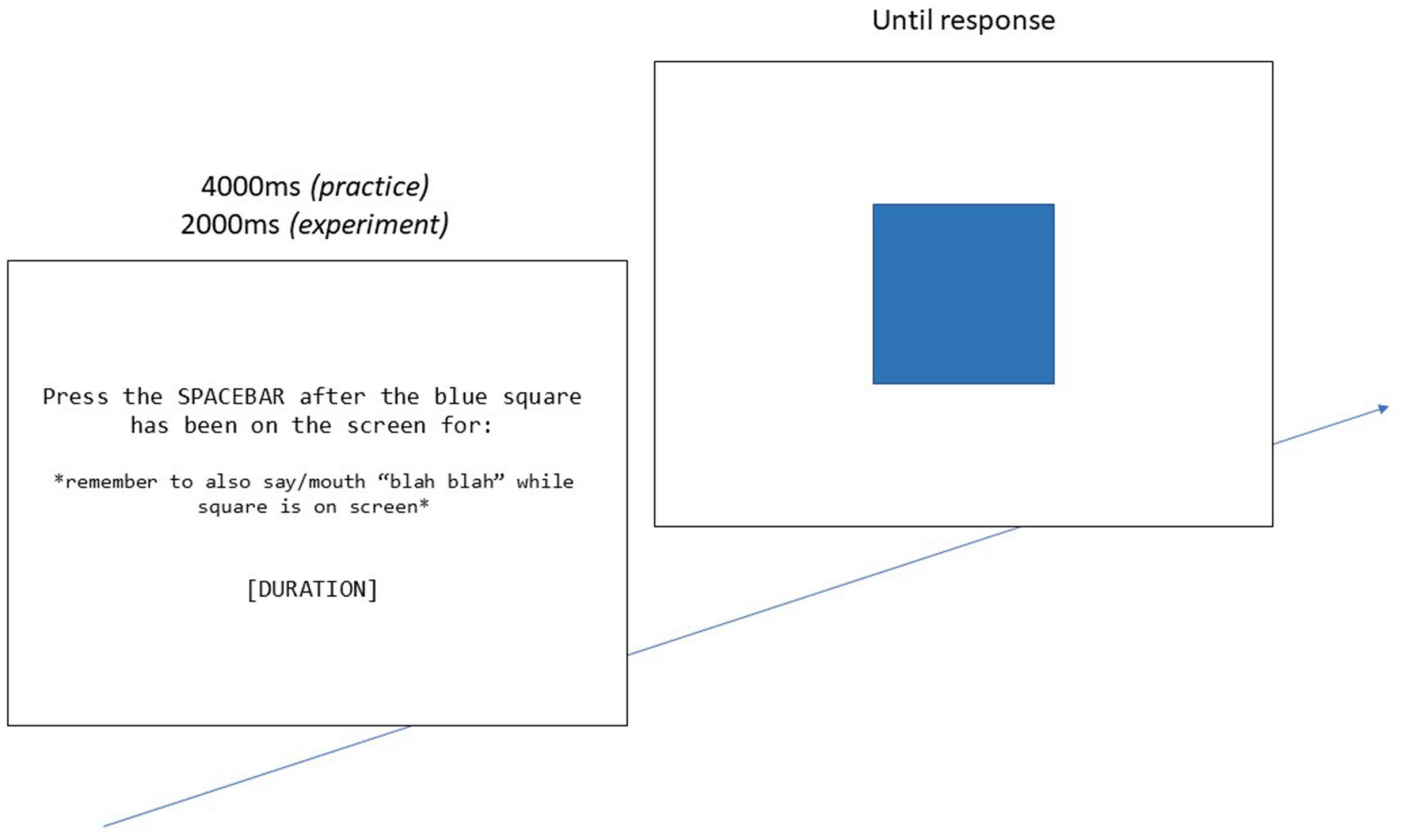
The “[DURATION]” is either 5 s, 8 s, 18 s, or 23 s. Each duration repeats 4 times for a total of 16 trials.

The time production paradigm (TPT) (Vanneste et al., 2016) used in this experiment utilized prospective time estimation and was used to measure one’s internal clock speed. The task began with a brief practice phase that was not included in final analyses. Participants were instructed to memorize the target duration of time (e.g., “5 s”) that appeared in the center of the screen. Then, a blue square would appear in the center of the screen and participants were asked to press the spacebar when they estimated the target duration to be over. They were also asked to engage articulatory suppression and prevent the use of counting strategies, as suggested by Vanneste et al. (2016). The procedure is illustrated in Figure 2.

The time production task was scored in terms of accuracy. For each trial, the subjective observed duration (SD) was divided by the objective target duration (OD). Values above 1 reflected overestimation of time, and values smaller than 1 reflected underestimation.

#### Statistical analyses

All analyses for both studies were conducted using IBM SPSS 28.0.1 (IBM Corp, 2021). Composite scores for the survey variables of interest were calculated and screened for normality. In both studies, the Stop Signal Task omission and commission errors were square root-transformed and age was log-transformed. In Study 2, survey time estimation was also log-transformed. Square root transformation rather than log was used for the first two variables because a large number of valid datapoints were 0 s (indicating no errors on the task).

Descriptive statistics for the survey and behavioral measures for both studies can be found in Supplementary Tables 8–10. Bivariate correlation analyses (both zero-order and controlling for age, gender, ethnicity, and the use of a Screen Time App to enter hours) were conducted before continuing to regressions. In Study 1, we also controlled for whether the participant took part in the E-Prime portion of the study. We performed exploratory analyses examining the associations between the variables of interest and individual media multitasking index scores, each media as a background activity, and individual foreground-background task combinations. Finally, due to the large number of items in the survey, we also conducted consecutive item correlation analyses and found no significant issues.

### Results

#### Media multitasking variables

All participants engaged in some level of media multitasking, and the sample average general media multitasking frequency (MMI) score was 3.06 (*SD* = 1.44, *range* = 0.12–9.71). The MMI was slightly skewed, but within acceptable limits (skew = 0.742, kurtosis = 1.142; Curran et al., 1996). The most time-consuming daily activity was speaking face-to-face with another person (*M_hours_* = 5.81, *SD* = 3.92), followed by using social media sites (*M_hours_* = 4.61, *SD* = 3.30), and the least was using print media (*M_hours_* = 0.95, *SD* = 1.27). Texting was the most common background activity (*M* = 43.47%, *SD* = 20.24), followed by listening to audio entertainment (*M* = 40.76%, *SD* = 21.28). Additional media use variable characteristics can be found Supplementary Table 8.

Over half of the sample reported multitasking during the survey portion of the study (*N* = 269; 55.20%) and 20.00% during the E-Prime portion. The most common form of media multitasking during the survey was listening to audio entertainment in the background (*N* = 52; 20.00% of in-study multitasking). An independent samples t-test showed that MMI score did not significantly vary between those who did and did not multitask during the survey portion [*t*(478) = −0.25, *p* = 0.80]. However, those who did media multitask during the E-Prime portion (*M*_MMIScore_ = 3.42) had a significantly higher MMI score than those who did not [*M*_MMIScore_ = 2.85; *t*(295) = −2.91, *p* = 0.004, Cohen’s *d* = 0.42]. There was also a weak association between in-study media multitasking during the survey and E-Prime portions (*χ*^2^ = 5.03, *p* = 0.025, *φ* = 0.13). Accounting for the control variables mentioned in *Statistical Analyses* did not notably change these associations. Using a logistic regression to control for the control variables and separately, to control for MMI still found a significant relation between the two in-task multitasking variables.

#### Cognitive control and mindfulness variables

Hypotheses 1 through 3 were examined using bivariate correlations and were partially supported (see Supplementary Table 11). Both in-study media multitasking measures, though not the general media multitasking frequency score, were significantly associated with a lowered total effortful control score. Those who were multitasking during the survey portion of the study also had significantly lower activation control [*t*(484) = 3.51, *p* < 0.001, Cohen’s *d* = 0.32] and attentional control scores [*t*(484) = 2.39, *p* = 0.018, Cohen’s *d* = 0.22], which may have implications for struggles with goal persistence and focus in those who tend to media multitask during academic tasks like research studies (RQ1). Self-reported inhibitory control correlated negatively with both MMI score (*r* = −0.11, *p* = 0.007) and with multitasking during the E-Prime portion of the study (H2), such that those who multitasked had lower inhibitory control scores [*M* = 27.60 vs. *M* = 29.28; *t*(297) = 2.16, *p* = 0.032, Cohen’s *d* = 0.31].

Mindfulness and its sub-facets were largely unrelated to media multitasking behavior. However, both MMI score and in-study multitasking during the survey correlated negatively with the nonjudging facet.

Accounting for control variables did not significantly alter the associations between the cognitive control variables. However, for the subsample of participants who completed the E-Prime portion of the study (*n* = 286), all relations between MMI score and the cognitive control variables became non-significant. In-study multitasking remained negatively associated with effortful control and some mindfulness subscales (see Supplementary Table 12).

Exploratory analyses as outlined in *Statistical Analyses* can be found in Supplementary material. No association was above *r* = 0.20, but some interesting patterns emerged. This may inspire future studies on immediate gratification and specific media combinations.

#### Time-related variables

The MMI score had a medium positive correlation with the CFC immediate consequences-focused subscale (*r* = 0.20, *p* < 0.001; H4; see Supplementary Table 14) and in-study media multitasking during the survey portion had a small negative correlation with the CFC future subscale (*r* = −0.09, *p* = 0.024), which indicates that media multitasking was associated with favoring immediate goals over long-term ones. Media multitasking during the survey portion was also associated with underestimating the time it took to complete the survey, which is in line with prior experimental research (*r* = −0.15, *p* < 0.001) (Xu and David, 2018).

Accounting for control variables did not significantly alter these relations. However, for the subsample of participants who completed the E-Prime portion of the study (*n* = 287; Supplementary Table 16), there were additional significant partial correlations between MMI and overestimating survey duration (*r* = 0.13, *p* = 0.026), and multitasking during the survey with CFC future (*r* = −0.14, *p* = 0.022) and immediate-focused (*r* = 0.13, *p* = 0.034) subscales, as well as a weak relation between these two subscales (*r* = −0.12, *p* = 0.046).

Exploratory analyses revealed that watching videos (*r* = 0.22, *p* < 0.001) and talking on the phone or video chatting (*r* = 0.22, *p* < 0.001) as background activities was associated with a greater immediate goal and reward focus.

#### Regression analyses

Before regression analyses, we calculated bivariate correlations between cognitive control, mindfulness, and the time-related variables (Table 2). The associations were in the expected directions. With the transformed variables, the regression met required assumptions.

**Table 2:** Summary of correlation analysis on cognitive control with time perspective variables (*N* = 298–487).

Variables	Survey time est. (Avg)	Time production task (average)	CFC future	CFC immediate
ATQ effortful control total	−0.01	0.06	0.21^***^	−0.24^***^
ATQ EC activation	−0.01	0.09	0.25^***^	−0.22^***^
ATQ EC attentional	0.03	0.01	0.06	−0.16^***^
ATQ EC inhibitory	−0.03	0.02	0.14^**^	−0.16^***^
FFMQ total	−0.05	0.07	0.20^***^	−0.23^***^
FFMQ observing	−0.05	0.09	0.32^***^	0.02
FFMQ describing	−0.01	0.17^**^	0.21^***^	−0.18^***^
FFMQ ActAware	−0.07	−0.03	0.05	−0.34^***^
FFMQ Nonjudge	−0.03	−0.08	−0.10^*^	−0.20^***^
FFMQ Nonreact	0.04	0.08	0.17^***^	0.09^*^
SST Go RT	−0.01	−0.01	−0.05	−0.01
SST omission	0.04	0.05	0.10	0.04
SST commission	0.05	−0.14^*^	−0.06	−0.02

FFMQ, Five Facet Mindfulness Questionnaire; ATQ, Adult Temperament Questionnaire; EC, Effortful Control; SST, Stop Signal Task; RT, reaction time; CFC, Considerations of Future Consequences scale. Two-tailed significance: *p < 0.05, **p < 0.01. ***p < 0.001.

Multiple regression analyses were conducted to determine the contribution of the sets of independent variables (i.e., demographics and control variables at step 1, cognitive control variables at step 2, and time perspective variables at step 3) to the variance in media multitasking. Two blockwise regressions were conducted: one which included both survey-derived and E-Prime task measures (Table 3) and one which included only survey measures (Supplementary Table 17).

**Table 3:** Summary of blockwise multiple regression analysis on media multitasking index score, in subsample who completed both portions of the study (*n* = 292).

	Step 1			Step 2			Step 3		
Variable	*B* (*SE*)	β	*p*	*B* (*SE*)	β	*p*	*B* (*SE*)	β	*p*
Constant	6.21 (3.02)		0.041	6.47 (3.32)		0.052	3.86 (3.25)		0.236
Screen Time App	−0.09 (0.17)	−0.03	0.600	−0.10 (0.17)	−0.03	0.572	−0.06 (0.16)	−0.02	0.699
Gender	0.20 (0.16)	0.08	0.196	0.16 (0.17)	0.06	0.333	0.16 (0.16)	0.06	0.321
Age	−2.71 (2.35)	−0.07	0.250	−2.88 (2.36)	−0.07	0.224	−3.00 (2.26)	−0.08	0.186
Ethnicity	0.05 (0.06)	0.05	0.401	0.07 (0.06)	0.07	0.271	0.06 (0.06)	0.06	0.324
FFMQ				0.20 (0.24)	0.06	0.397	0.33 (0.23)	0.10	0.148
ATQ effortful control				−0.14 (0.15)	−0.07	0.361	−0.08 (0.15)	−0.04	0.571
SST go reaction time				−0.00 (0.00)	−0.03	0.677	−0.00 (0.00)	−0.02	0.723
SST omission				0.09 (0.10)	0.06	0.334	0.09 (0.09)	0.06	0.324
SST commission				0.12 (0.11)	0.07	0.274	0.14 (0.11)	0.08	0.193
Survey time estimation							0.46 (0.17)	0.16^**^	0.007
TPT time estimation							−0.40 (0.37)	−0.06	0.272
CFC future							0.02 (0.01)	0.09	0.112
CFC immediate							0.05 (0.01)	0.26^***^	<0.001
*F*	0.95		0.435	0.92		0.512	3.10		<0.001

FFMQ, Five Facet Mindfulness Questionnaire; ATQ, Adult Temperament Questionnaire; SST, Stop Signal Task; TPT, Time Production Task; CFC, Considerations of Future Consequences Scale; β, standardized betas. *p < 0.05, ***p < 0.001. Step 1: *R*^2^ = 0.01, Adjusted *R*^2^ = −0.00; Step 2: *R*^2^ = 0.03, Adjusted *R*^2^ = −0.00, *R*^2^ Δ = 0.02, *p* = 0.068; Step 3: *R*^2^ = 0.13, Adjusted *R*^2^ = 0.09, *R*^2^ Δ = 0.10, *p* < 0.001.

The largest significant contribution of variance in media multitasking frequency (as measured by the MMI) came from having an immediate time perspective (*β* = 0.26, *p* < 0.001). Survey time overestimation was also significant (*β* = 0.16, *p* = 0.007). Adding the third step to the regression analysis, which contained the CFC subscales, survey time estimation, and TPT estimation resulted in a significant model [*F*(13, 279) = 3.10, *p* < 0.001]. The variables combined to explain 8.5% (adjusted *R^2^*) of the variance in Media Multitasking Score. A post-hoc sensitivity analysis^1^ indicated that a sample size of 292 was large enough to detect an effect size of f^2^ = 0.04 when *α* error probability was set to 0.05 and power to 80%.

### Discussion

We hypothesized that greater effortful control in general (H1) and inhibitory control in particular (H2) would be associated with less media multitasking behavior. We also aimed to examine the relation between media multitasking and activation control (RQ1). Hypothesis 1 was not supported using the MMI survey, but it was for in-study multitasking behavior. Self-reported inhibitory control was negative correlated with MMI, but the behavioral measures were not, partially supporting Hypothesis 2. In-study multitasking was also correlated with activation control issues. We hypothesized that greater trait mindfulness would be associated with lower levels of media multitasking (H3). We also aimed to examine the relation between media multitasking and the subfacets of mindfulness (RQ2). Hypothesis 3 was not supported using the overall mindfulness measure, but there were associations with the nonjudging mindfulness facet: those who media multitask more in general and who were multitasking during the survey in particular tend to assign labels (e.g., “good,” “bad”) to their thoughts and emotions, rather than leaving them unjudged.

We hypothesized that having an immediate-focused time perspective (H4) would be associated with greater media multitasking, and that in-study multitasking would be associated with time passage underestimation on the survey portion (H5). Hypothesis 4 was supported, and an immediate-focused time perspective was the strongest predictor of MMI in a regression analysis. Hypothesis 5 was also supported. Interestingly, media multitasking frequency was associated with overestimating the time it took to complete the survey, but actually multitasking during the survey was associated with time underestimation. Perhaps the survey did feel like it was taking too long, but multitasking with a pleasant activity during it invoked a feeling of “time flying.”

We also aimed to examine whether time under- or overestimation as measured by the Time Production Task would be associated with more media multitasking behavior (RQ3). The performance assay measure of time under- or over-estimation was not associated with media multitasking, which supports the contextual/typical versus optimal performance divide that is also seen in cognitive control studies (Parry and Le Roux, 2021).

Our hypotheses were partially supported, though the correlation coefficients and amount of variance explained in the regression were modest. This may be due in part to the online nature of data collection; there may have been a large amount of variability in participants’ surroundings while completing the study. We addressed this possibility in Study 2.

## Study 2

A second study was conducted to replicate the results of Study in a controlled laboratory setting. We added a measure of delay discounting (Money Choice Questionnaire; Kirby and Maraković, 1996) to probe immediate gratification further and replicate past findings (Schutten et al., 2017). We expected a preference for immediate reward to be positively associated with media multitasking behavior (H6). The dataset for Study 2 can be found here: https://osf.io/wdusj/?view_only=d13b624b0e2a465a854b7075afdd8b58.

### Materials and methods

#### Participants

A total of 383 participants were recruited from University 2’s Psychology research pool and compensated with course credit. Two were removed due to failing at least two of the three attention checks, leaving the final sample with 381 participants. The demographics information for Study 2 is listed in Table 1. Our power analysis^2^ was based on a blockwise regression outcome from Study 1 that did not use transformed variables (recommended *N* = 375), so we decided to conduct sensitivity analyses for the hypotheses in Study 2 as well (see Supplementary material).

#### Procedure

The procedures for Study 2 were largely the same as Study 1, except the components were completed in person in a research lab setting. There was also one additional measure in the survey portion and the Time Production Task was removed from the E-Prime portion. The survey took approximately 38 min and the E-Prime task took approximately 11 min to complete.

#### Measures

The survey measures were the same as in Study 1 with one addition. There were no major differences between the measured descriptive statistics in the two samples, though Study 1 had slightly higher media use times, media multitasking scores, and survey estimation times.

##### Money Choice Questionnaire

The Money Choice Questionnaire (MCQ-21; Kirby and Maraković, 1996) is a 21-item measure of delay discounting. Participants are asked to choose between a smaller monetary reward tonight or a larger reward in the future, resulting in a *k* value calculated via an Excel calculator (Kaplan et al., 2016). A larger *k* signifies a stronger preference for smaller short-term instead of larger long-term rewards. The *k* value is often quite right-skewed, so it is common practice to use a log-transformed value of *k* instead.

### Results

#### Media multitasking variables

The sample average general media multitasking frequency (MMI) score was slightly lower, *M* = 2.86 (*SD* = 1.21, range = 0.42–8.15) compared to Study 1. The MMI was slightly skewed, but still within acceptable limits (skew = 0.988, kurtosis = 1.402). Approximately half (*n* = 174; 45.70%) of participants multitasked during the survey and 16.50% (*n* = 63) during E-Prime. The most common form of in-study multitasking was listening to audio both during the survey (35.60% of those multitasking, or 16.30% of full sample) and E-Prime (80.90% of those multitasking, or 13.40% of full sample).

General media multitasking frequency as measured by the MMI correlated with in-study media multitasking during both the survey (*r* = 0.15, *p* = 0.004) and the E-Prime task portions (*r* = 0.10, *p* = 0.043). An independent samples *t*-test showed that those who multitasked during the survey (*M*_MMI_ = 2.87) had a significantly higher MMI score compared to those who did not [*M*_MMI_ = 2.51; *t*(379) = −2.93, *p* = 0.004, Cohen’s *d* = −0.30]. The results were similar for multitasking during the E-Prime portion [*t*(376) = −2.03, *p* = 0.04, Cohen’s *d* = −0.28; *M*_MMIYes_ = 2.96, *M*_MMINo_ = 2.62]. There was also a moderate association between in-study media multitasking during the E-Prime and survey portions [*r* = 0.36, *p* < 0.001; *χ*^2^(1) = 49.30, *p* < 0.001, *φ* = 0.36]. Accounting for control variables, and additionally for MMI in the relation between the two in-study multitasking variables, made these associations slightly larger.

#### Cognitive control variables

Media multitasking frequency was significantly negatively related to overall self-reported effortful control (*r* = −0.12, *p* = 0.025) but not inhibitory control (*r* = −0.10, *p* = 0.064), partially supporting our hypotheses (though inhibitory control was in the predicted direction). Multitasking was also associated with omission errors on the Stop Signal Task (*r* = 0.12, *p* = 0.022; see Supplementary Table 19). Multitasking during the survey was also associated with lower self-reported inhibitory control after accounting for controls (*r* = −0.11, *p* = 0.033). None of the media multitasking measures were significantly associated with activation control (RQ1).

Media multitasking frequency had small but significant correlations with all facets of mindfulness except Observing. The strongest relations were between both MMI score (*r* = −0.20, *p* < 0.001) and in-study multitasking during the survey (*r* = −0.19, *p* < 0.001) and the acting with awareness facet: those who tended to multitask more in general and during the survey in particular also indicated less active awareness in their daily lives, which is in line with previous research (Ralph et al., 2014). The association between in-study multitasking during the survey and lower acting with awareness became stronger when accounting for control variables (*r* = −0.21, *p* < 0.001).

#### Time-related variables

MMI score was associated with a preference toward short-term goals/rewards, measured via both the CFC (*r* = 0.14, *p* = 0.007) and the MCQ (*r* = 0.16, *p* = 0.002; see Supplementary Table 20). Multitasking during the survey was also associated with a higher CFC Immediate score (*r* = 0.14, *p* = 0.008), supporting Hypothesis 4, though not with survey time underestimation, counter to Hypothesis 5 (*r* = 0.05, *p* = 0.29). Those with higher MMI scores also *over*estimated the time they took to complete the survey portion of the study. Most of these associations got slightly stronger after controlling for demographics and the use of a Screen Time App to report media time. The video subscale of the MMI (*r* = 0.24, *p* < 0.001) and texting as a background activity (*r* = 0.19, *p* < 0.001) also correlated positively with steeper delay discounting. Additional individual activity analyses are listed in Supplementary material.

#### Regression analyses

Before regression analyses, we calculated bivariate correlations between the cognitive control, mindfulness, and the time-related variables (Table 4). With the transformed variables, the regression met required assumptions.

**Table 4:** Summary of correlation analysis on cognitive control with time perspective variables (*N* = 381).

Variables	log survey time Est. (Avg)	CFC future	CFC immediate	MCQ log *k*
ATQ effortful control total	0.02	0.32^***^	−0.40^***^	−0.09
ATQ EC activation	0.01	0.31^***^	−0.37^***^	−0.14^**^
ATQ EC attentional	0.02	0.22^***^	−0.33^***^	0.01
ATQ EC inhibitory	0.02	0.24^***^	−0.27^***^	−0.05
FFMQ total	−0.07	0.28^***^	−0.37^***^	−0.04
FFMQ observing	−0.09	0.31^***^	−0.02	0.01
FFMQ describing	−0.04	0.20^***^	−0.28^***^	−0.05
FFMQ ActAware	−0.06	0.19^***^	−0.43^***^	−0.03
FFMQ Nonjudge	0.02	−0.04	−0.20^***^	0.01
FFMQ Nonreact	−0.03	0.24^***^	−0.11^*^	−0.08
SST Go RT	−0.00	0.06	−0.04	−0.10^*^
sqrt SST Omission	0.13^*^	0.04	−0.05	−0.03
sqrt SST Comm	0.05	−0.03	−0.10	−0.04

FFMQ, Five Facet Mindfulness Questionnaire; ATQ, Adult Temperament Questionnaire; EC, Effortful Control; SST, Stop Signal Task; RT, reaction time; CFC, Considerations of Future Consequences scale; MCQ, Money Choice Questionnaire. Two-tailed significance: *p < 0.05, **p < 0.01, ***p < 0.001.

A blockwise regression was conducted (Table 5). The final model explained 9.70% of the variance in media multitasking index score. CFC Immediate had a smaller effect size (*β* = 0.13, *p* = 0.048 compared to *β* = 0.212, *p* < 0.001) compared to Study 1. In this study, having a future time perspective also emerged as a positive predictor of media multitasking frequency. Adding the delay discounting score (MCQ log *k*) explained additional 2.00% of variance, and it was the largest effect sizes at Step 4 (H6; *β* = 0.18, *p* < 0.001). A sensitivity analysis^3^ indicated that a sample size of *N* = 381 was large enough to detect an effect of f^2^ = 0.02. The effect size at Step 4 was f^2^ = 0.02.

**Table 5:** Summary of Blockwise Multiple Regression Analysis on Media Multitasking Index Score (*n* = 381).

	Step 1			Step 2			Step 3			**Step 4**		
Variable	*B* (SE)	β	*p*	*B* (SE)	β	*p*	*B* (SE)	β	*p*	*B* (SE)	β	*p*
Constant	7.90 (3.12)	0.012		9.16 (3.31)	0.006		6.60 (3.42)		0.055	8.06 (3.42)		0.019
STA	0.13 (0.14)	0.05	0.356	0.16 (0.14)	0.06	0.232	0.19 (0.14)	0.07	0.172	0.20 (0.13)	0.07	0.144
Gender	0.17 (0.12)	0.07	0.157	0.15 (0.21)	0.07	0.225	0.16 (0.12)	0.07	0.195	0.19 (0.12)	0.08	0.119
Age	–4.39 (2.43)	−0.09	0.072	–4.17 (2.43)	–0.09	0.087	–3.17 (2.45)	–0.07	0.197	–3.92 (2.43)	–0.08	0.108
Ethnicity	−0.02 (0.06)	−0.02	0.755	–0.04 (0.06)	0.03	0.530	–0.04 (0.06)	–0.03	0.530	–0.03 (0.06)	–0.03	0.568
Financial Stress	0.01 (0.00)	0.17^***^	<0.001	0.01 (0.00)	0.14^**^	0.007	0.01 (0.00)	0.14^**^	0.018	0.00 (0.00)	0.09	0.077
FFMQ				–0.01 (0.01)	–0.12^*^	0.047	–0.01 (0.01)	–0.10	0.121	–0.01 (0.01)	–0.10	0.088
ATQ EC				0.00 (0.01)	0.00	0.976	–-0.00 (0.01)	–0.01	0.911	0.00 (0.01)	–0.00	0.956
SST Go RT				–0.00	–0.02	0.753	–0.00 (0.00)	–0.02	0.709	0.00 (0.00)	–0.01	0.893
SST Omission				0.22 (0.1)	0.14^*^	0.022	0.19 (0.10)	0.12^*^	0.042	0.20 (0.09)	0.12^*^	0.038
SST Commiss				–0.12 (0.09)	–0.07	0.178	–0.11 (0.09)	–0.07	0.229	–0.11 (0.09)	–0.07	0.224
STE (Avg)							1.27 (0.53)	0.12^*^	0.018	1.28 (0.53)	0.12^*^	0.12^*^
CFC Future							0.01 (0.10)	0.12^*^	0.046	0.02 (0.01)	0.13^*^	0.028
CFC Immediate							0.02 (0.01)	0.13^*^	0.048	0.02 (0.01)	0.13	0.087
MCQ log *k*										0.31 (0.10)	0.16^**^	0.003
*F*	4.14		0.001	3.20	<0.001		3.38	<0.001		3.86		<0.001

STA = Use of Screen Time App, FFMQ = Five Facet Mindfulness Questionnaire, ATQ EC = Adult Temperament Questionnaire Effortful Control, SST = Stop Signal Task, STE = Survey Time Estimation, CFC = Considerations of Future Consequences Scale, MCQ = Money Choice Questionnaire. β = standardized betas. * *p* < 0.05. ** *p* < 0.01. *** *p* < 0.001. Step 1: *R^2^* = 0.05, Adjusted *R^2^* = 0.04; Step 2: *R^2^* = 0.08, Adjusted *R^2^* = 0.06, *R^2^* Δ *=* 0.03, *p* = 0.05; Step 3: *R^2^* = 0.11, Adjusted *R^2^* = 0.08, *R^2^* Δ *=* 0.03, *p* = 0.011 Step 4: *R^2^* = 0.131, Adjusted *R^2^* = .10, *R^2^* Δ *=* 0.02, *p* = 0.003.

The importance of context was highlighted by completing additional exploratory regressions, with the media multitasking index subscales and individual media-as-background-activity as dependent variables. The same predictors explained 16.60% of the variance in playing video games while multitasking with other tasks. This result was mostly driven by gender—traditionally, men have been shown to play video games more than women (Greenberg et al., 2010). The effect size of the CFC immediate subscale was also significant, perhaps because video games incentivize short-term achievement goals. The predictors explained 13.50% of the variance in using social media as a background task but only 4.30% variance in using audio in the background. Social media use requires more modalities (i.e., visual, auditory, and tactile) than audio, so using it in the background causes greater interruption. This might be associated with more failures in cognitive control or a short-term focused time perspective.

#### Discussion

As in Study 1, we hypothesized that greater effortful control in general (H1) and inhibitory control in particular (H2) would be associated with less media multitasking behavior. We predicted that greater trait mindfulness would be associated with lower levels of media multitasking (H3). We also aimed to examine the relation between media multitasking and the subfacets of mindfulness (RQ2). Hypotheses 1, 2, and 3 were supported. However, H2 was only supported for the performance assay measure of inhibitory control—potentially because the sample size was not large enough to detect the significance of the small effect in the self-report measure. None of the media multitasking measures were significantly associated with activation control (RQ1). Acting with awareness was the mindfulness facet with the strongest associations with media multitasking, similar to prior research, though there were also associations with other facets (RQ2).

We hypothesized that having an immediate-focused time perspective (H4) would be associated with greater media multitasking and that in-study multitasking would be associated with time passage underestimation on the survey portion (H5). We also expected media multitasking behavior to be positively associated with an MCQ score that indicates favoring short-term rewards (H6). Hypotheses 4 and 6 were supported, but H5 had a null effect, as in the both-portions subsample of Study 1. Survey time *over*-estimation emerged as a significant predictor of media multitasking frequency. The results largely replicated Study 1 and supported most of our hypotheses.

## General discussion

The purpose of this study was to investigate variables that may underlie the immediate gratification media multitasking grants—cognitive control, mindfulness, and time perspective and perception—using Hofmann et al. (2009) and Fujita (2011) views of self-control. We conducted two studies using two different student samples and a multi-method approach. Having a present-focused time perspective and favoring a short-term decision-making style, emerged as the strongest predictors of media multitasking frequency. Additional smaller associations with specific aspects of mindfulness, cognitive control, and time estimation were also found. Overall, our findings suggest that a *preference* for immediate reward might outweigh cognitive control *ability* when predicting media multitasking behavior, supporting Fujita (2011) dual-motive model.

The studies also contribute to the literature methodologically: We used the Time Production Paradigm in the context of media multitasking for the first time, investigated activation control and additional mindfulness facets, and replicated limited prior findings on immediate time perspective and time estimation in the context of media multitasking. We also utilized in-study multitasking as a variable and put more focus on the MMI subscales and media used as background activities. Finally, in Study 1, we investigated these phenomena in a diverse student sample.

### Cognitive control

We used Hofmann et al. (2009) dual-process model of self-control to guide our hypotheses. We expected inhibitory control, derived from the Stop Signal Task (System 1) and the Adult Temperament Questionnaire—Effortful Control scale (System 2), to be negatively related to media multitasking frequency. We also expected overall effortful control and overall mindfulness, measured by the Five Factor Mindfulness Questionnaire—another System 2 measure, to be negatively associated with media multitasking frequency. Finally, we posed research questions about the relations between media multitasking, the activation control subscale of effortful control, and individual mindfulness subscales.

The hypotheses pertaining to cognitive control were partially supported. Although the media multitasking score (MMI) did not significantly correlate with either effortful control (H1) or total mindfulness (H3) scores in Study 1, it did in Study 2. Although the MMI has never been used in conjunction with the ATQ-EC or FFMQ, prior studies did find medium to strong correlations between the measure and other self-report measures of self-regulation and the acting with awareness facet of mindfulness (Ralph et al., 2014; Sansevere and Ward, 2021; Yildirim and Dark, 2018). Those who have better self-regulation ability and attentional control are better able to focus their attention on one media stream at a time. The results of the present study indicate that mindfulness as a whole, not just its attentional control aspect, may be relevant to media multitasking behavior, though this relation may not be equally strong in all populations.

In both studies, general effortful control was also negatively associated with in-study multitasking. Interestingly, we found no correlation between media multitasking score and self-reported inhibitory control (H2b) in either the subsample of participants who completed the E-Prime portion in Study 1 or across the entire sample of Study 2. A recent meta-analysis (Parry and Le Roux, 2021) indicated that it is common to see small to null associations between media multitasking and performance assay (System 1) measures of inhibitory control, as replicated by the Stop Signal Task measure results. However, the meta-analysis also reported small to medium negative effects between self-reported inhibitory control (System 2) and MMI score, so the null result is unexpected. However, this particular inhibitory control scale has not been used in a media multitasking context before, and may be probing a slightly different construct than the traditional measures. Additionally, the low reliability of the measure, particularly in Study 1, might have suppressed the effect.

Alternatively, as this was a small effect (*r* = 0.11) it is possible that the Study 1 subsample of participants who completed both parts of the study (*N* = 300) and the Study 2 sample (*N* = 381) were underpowered to detect a small, but significant effect. A structural regression model using a combined dataset (*N =* 868; see Supplementary material) found an association of *r* = −0.18 (*p* = 0.006). Inhibitory control is implicated in being better able to filter out distractions and control responding to the environment, so those who media multitask more may have difficulties controlling their responses. For example, they may reach for their phone immediately when they get a notification, no matter what their main task is at the time. More research is needed to say whether the lowered inhibitory control causes more media multitasking, whether the reverse is true, or whether it is a bidirectional relationship as is common in media research (Valkenburg et al., 2016).

Regarding RQ1, there was an association between activation control and multitasking during the survey portion in Study 1. Activation control is key in overriding avoidance and procrastinatory behavior, which also aligns with prior research that has found positive associations between media multitasking tendency and avoidance-based coping (Evans and Rothbart, 2007; Shin and Kemps, 2020). Future research should continue to examine multitasking with media for the purpose of self-regulation. Interestingly, activation control was not associated with in-study multitasking in Study 2. This may be because in Study 1, participants used a secondary media stream to compensate for their lower activation control, stay on task, and avoid pursuing other goals in their vicinity (e.g., talking with friends, doing homework). Many participants were listening to music, which is often used with the intention of improving focus (de la Mora Velasco et al., 2023). Since Study 2 took place in a lab setting, the access to additional activity goals was much lower—participants had to complete the study before doing anything else. Under these conditions, having lower activation control would then not be associated with using secondary media to stay on-task.

Regarding RQ2, both the media multitasking score and multitasking during the survey portion of the study correlated negatively with the nonjudging of inner experience facet of mindfulness in both studies. This phenomenon is often linked to depression and anxiety via increased rumination (Barcaccia et al., 2019). As mentioned above, prior research has found that media multitasking frequency is associated with using more avoidance-based coping (Shin and Kemps, 2020). It is possible that individuals who judge their thoughts and feelings more may turn to multiple media streams to block out thinking and suppress rumination. Future research should probe this relation further, to see if multitasking with media is associated with more rumination, if it is a strategy used to block out thoughts or emotions one judges as negative, and what long-term consequences might be.

### Time-related variables

We used Fujita (2011) dual-motive model to inform our hypotheses about time perspective and used time perception as a related measure of System 1 self-control. The hypotheses pertaining to time were partially supported. We predicted that more media multitasking would be associated with an immediate-focused time perspective (H4) and that in-study multitasking would be associated with underestimation of time (H5). We also posed a question about the relation between media multitasking and a time estimation performance assay (RQ3). In Study 2, we also predicted that more media multitasking behavior would be associated with steeper delay discounting (preferring short-term over long-term rewards; H6). The CFC immediate consequences-focused subscale and media multitasking tendency (i.e., MMI score) had a small-medium positive correlation in Study 1 and a small correlation in Study 2 (H4). In both Study 2 and the E-Prime subsample of Study 1, the immediate-focused subscale was also positively associated with multitasking during the survey. Even with the addition of all variables of interest in the multiple regression analyses in Study 1, the CFC immediate subscale was the strongest predictor of MMI. CFC immediate also correlated at above *r* = 0.2 with watching video content and talking to others on the phone as background activities, and combining gaming with face-to-face conversation and social/non-social site browsing. Finally, in-study multitasking was also positively associated with having an immediate-focused time perspective, which is in line with Labăr and Ţepordei (2019) findings.

These results may indicate that people who multitask more are attempting to complete *all* of their goals at once, in order to feel the reward of a sense of accomplishment as quickly as possible. They might also be indicative of a problem managing goal conflict, persistence, and disengagement (Brandstätter and Bernecker, 2022), generally having difficulty prioritizing their goals. Effects of a similar size have been found in studies examining the relations between present-focused time perspective and problematic or addictive media use (Kim et al., 2017; Settanni et al., 2018). Although more habitual media users tend to have more problematic use *and* tend to multitask with media more, the two are distinct concepts. Future studies should investigate the role time perspective plays in media addiction and media multitasking behaviors and assess any differences between the two. Comparisons can also be made with real-time media multitasking during specific situations, like in class or during leisure time, instead of measuring real-time multitasking during the study tasks retrospectively. Future research should also explicitly ask participants about their goals for a media multitasking session. To our knowledge, the present study was the first to examine the relation between individual differences in time perspective and media multitasking frequency, so further replication in different samples is necessary.

In Study 2, the immediate subscale of the CFC played a smaller, but still significant, role. In the regression model, the future subscale of the CFC was actually a stronger predictor than the immediate. The strongest relation between CFC future and a media multitasking subscale was with multitasking while doing homework, which may have driven the effect. This points to the complex nature of media multitasking. It may be used for entertainment and avoidance coping, or for productivity. Future research should investigate the role of time perspective specific media multitasking situations and combinations.

There were conflicting findings regarding time estimation (H5, RQ3). The performance-based assay examining time estimation ability had null relations with media multitasking, mirroring prior small or null relations between media multitasking and performance-based assays of cognitive control (Parry and Le Roux, 2021). In-study media multitasking during the survey in Study 1 correlated weakly but significantly with underestimating the amount of time it took to complete the survey. However, in Study 2 and in the E-Prime subsample of Study 1, media multitasking frequency (MMI) was actually positively associated with *over*-estimating survey completion time. Both over- and under-estimation have been associated with impulsivity and cognitive control deficits. As the present and prior studies indicated, media multitasking frequency is associated with self-control difficulties—perhaps issues with time estimation are just one manifestation of these self-control differences.

It should be noted that issues with time perspective, time estimation, and increased media multitasking are also associated with Attention-Deficit Hyperactivity Disorder (ADHD) (Ptacek et al., 2019; Fisher et al., 2023), so the diagnosis may act as a confounding variable. We conducted our regressions again with ADHD diagnosis as a control variable, and although it explained extra variance in MMI, time overestimation and an immediate time perspective remained a similar strength and significance (see Supplementary material). Future investigations should examine the role of the diagnosis as well. Future research should also consider participants’ proneness to boredom and preference for multitasking in general (i.e., polychronicity) as potential mechanisms for this issue with time estimation. Those who are more easily bored or prefer to interface with multiple tasks at once might feel the pressure of a singularly-focused task like a research study more than others. Frequency of media multitasking and polychronicity have a medium meta-correlation (Howard and Cogswell, 2023), so adding measures of polychronicity to future studies may inform additional conclusions.

Finally, Hypothesis 6, that media multitasking would be associated with a preference for immediate reward on a delay discounting task, was supported. This is in line with prior research done by Schutten et al. (2017). As with immediate-focused time perspective, it is possible that some individuals multitask because using multiple media magnifies the immediate feeling of reward they can attain. Future research should examine connections between the reward signals and media multitasking using experimental methods.

### Integrating cognitive control and time

Both guiding theories were supported, though the findings pointed to Fujita (2011) time motivations being more relevant to media multitasking behavior. Measures of both System 1 and System 2 self-control predicted media multitasking frequency, though the associations were null to small for the traditional System 1 measures. A focus on immediate rewards emerged as the most influential association with media multitasking behavior. Issues with time perception (i.e., under- and overestimating time passage) played a role as well. Overall, the self-control ability and tendency measures correlated in the expected ways with the time variables. This lends support to both a susceptibility (i.e., a present-focused time perspective leads to lower self-control ability) and buffering (i.e., a future-focused time perspective acts as a buffer against low self-control; Joireman et al., 2008) hypothesis. Fittingly, a longitudinal study found a reciprocal relation between future time perspective and self-control (Hao et al., 2024). Future research should investigate the role habitual media multitasking may play in this relation. It may increase as self-control decreases, or decrease if the individual undertakes goal-setting training and adopts a more future-focused time perspective. Alternatively, the media multitasking frequency may remain the same but the specific media combinations may change. For example, switching to listening to instrumental background music while working instead of music with lyrics.

As advised by a reviewer, we probed some interactions between time perspective, self-control, and media multitasking further. Both the acting with awareness facet of the FFMQ and the attentional control subscale of the ATQ-EC moderated the relation between having a present-focused time perspective and media multitasking frequency, such that the association was stronger at higher levels of attentional control. Those who were more present-oriented and better able to focus their attention also media multitasked more. This was counter to findings that those who multitask more actually have worse multitasking ability (Ophir et al., 2009). However, these were both self-report measures, and those who multitask more often report being able to multitask better—and if they are also more immediate-focused, they are more likely to *want* to attain more immediate reward via multiple media use. No other aspects of self-control moderated the relation between time perspective and media multitasking frequency.

A prior study also found that an internal attentional style more than an external one moderated the association between a time perspective and increased social networking addiction (Miceli et al., 2021). The acting with awareness and attentional control scales used here did not differentiate between internal and external attentional focus, but future research should investigate these variables together in the context of media multitasking.

Overall, examining time-related variables alongside more typical measures of self-control added important information about the relation between immediate gratification and media multitasking.

### Limitations and research implications

Although the current study contributes to the understanding of media multitasking, limitations exist. Participants in Study 1 were allowed to participate in the study via their personal devices in any setting, which may have added a significant amount of noise to our data. Additionally, although these studies focused on cognitive control, mindfulness and some time-related variables, many other predictors of multitasking, like polychronicity, motivations for use, availability of time and access to devices, and others may have stronger effects. The data were collected using a cross-sectional design, which limits drawing conclusions about the direction of effects. Some of the data were also collected using self-report questionnaires. Some of the measures used had low reliabilities and range restriction, which may have suppressed effect sizes. Finally, the studies were conducted using undergraduate student samples and were not balanced in gender (predominantly women) or ethnicity (largely Latine in Study 1 and White in Study 2), so cannot be generalized to other populations.

In addition to being a limitation, the last point can also be regarded as a strength of Study 1, as much of prior media use research from the United States and Europe has been conducted with primarily White samples. The two studies together also constitute a strength, as some results replicated in two student samples that were vastly different in terms of geographic location, ethnicity, and background. It is promising that some findings, like the relations between media multitasking and a present-focused time perspective, appear across studies and sample demographics. However, the fact that some relations did not replicate opens questions. Future studies should continue to examine how people’s individual differences and similarities interact with their media use habits.

Both general media multitasking tendencies and real-time in-study multitasking had modest associations with both self-report *and* behavioral measures. Future studies should continue to take a multi-method approach while investigating media multitasking in more specific, ecologically valid, situations. Time-sampling or longitudinal research of media multitasking habits, and how they interact with trait aspects of immediate gratification, would paint a more complete picture.

## Conclusion

Although media multitasking has become almost impossible to avoid in daily living, there are subtle differences in its execution: from the number of devices and activities one can comfortably juggle, to its primary purpose, to the way it makes one feel. Even though the baseline level of media multitasking appears to be increasing as technology becomes more ubiquitous and easier to access, there is still individual variation. This study examined the role of cognitive control, mindfulness, and time-related variables including time perspective and perception in media multitasking behavior using data from two samples, novel measures, and a multi-method approach. Relations emerged between media multitasking and a preference for short-term over long-term goals, as well as procrastinatory tendencies. In all, it appears that a preference for immediate rewards plays a larger role than the ability to inhibit immediate gratification, when examining media multitasking behavior. Future research will need to examine potential mechanisms for these effects.

## Data Availability

The dataset for Study 1 presented in this article is not readily available because such a possibility was not listed in the IRB protocol or informed consent, and participants cannot be contacted now for permission. Requests to access the datasets should be directed to Alena Rogojina at rogojina@iastate.edu. The dataset for Study 2 is available at: https://osf.io/wdusj/?view_only=d13b624b0e2a465a854b7075afdd8b58.

## Footnotes

^1^*F*-tests: Linear Multiple Regression (Fixed model, R^2^ increase). Alpha error prob. = 0.05, power = 0.8, total sample size = 292, number of tested predictors = 4, total number of predictors = 14. With *R*^2^ *Δ = 0*.10, and *R*^2^ = 0.13, the total effect size from Step 3 was f^2^ = 0.11.

^2^*F*-tests: Linear Multiple Regression (Fixed model, R^2^ increase). Effect size f^2^ = 0.0447, alpha error prob. = 0.05, power = 0.9, number of tested predictors = 5, total number of predictors = 14.

^3^*F*-tests: Linear Multiple Regression: Fixed Model, R^2^ increase. Alpha error prob. = 0.05, power = 0.8, total sample size = 381, number of tested predictors = 1, total number of predictors = 14.
