# Supplementary material for: The importance of time perspective in media multitasking behavior
Source: Front Psychol. 2025 Oct 31;16:1654790. doi: 10.3389/fpsyg.2025.1654790 (PMC12615458; doi:10.3389/fpsyg.2025.1654790)
Supplement: Supplementary file 1 [file Supplementary_file_1.docx]

# Supplementary Materials

## Media Multitasking Index Calculation Example

Original explanation from Ophir et al. (2009):

To create the MMI, we assigned numeric values to each of the matrix responses as follows: “Most of the time” (=1), “Some of the time” (=0.67), “A little of the time” (=0.33), and “Never” (=0). For each primary medium, we summed the responses. This resulted in a measure of the mean number of other media used while using each primary medium. To account for the different amounts of time spent with each medium, the MMI was created by computing a sum across primary media use weighted by the percentage of time spent with each primary medium. This, the index is an indication of the level of media multitasking the participant is engaged in during a typical media-consumption hour. In summary, the formula is as follows:


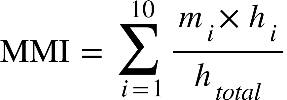


Where *m*_i_ is the number of media typically used while using primary medium *i*, *h_i_* is the number of hours per week spent using primary medium *i* and *h*_total_ is the total number of hours per week spent with all primary media.

Example

First, participants entered the number of hours they spent doing each activity on a typical day (modifying week to day is common when using the MMI). Say that all their activities added up to 16 hours per day. For example, doing homework for 2 hours a day. If a participant marked that they combined homework with print media 20% of the time, with face-to-face conversation 50% of the time, with texting 30% of the time, with using social media 10% of the time, with non-social sites 10% of the time, with talking on the phone 5% of the time, with listening to audio content 80% of the time, with watching video content 20% of the time, with playing games 0% of the time, and with studying for another class 10% of the, the equation would be as follows:

$$MMI\left( Homework \right)=\frac{\left( .20+.50+.30+.10+.10+.05+.80+.20+.10 \right)*2}{16}$$

Then, do the same for each activity and add them all up to get the final overall MMI.

## Factor Analyses—Studies 1 and 2

Study 1

*Adult Temperament Questionnaire-Effortful Control (ATQ-SF; Evans & Rothbart, 2007)*

The ATQ-SF as a whole and the EC subscale have been used frequently within student, community, and clinical samples. The EC subscale has an acceptable level of internal consistency (α = .77 – .83(Fosco et al., 2012), though that of the sub-facets tends to vary (α = .42 – .74; (De Panfilis et al., 2013; Kahn et al., 2018). EC has been associated with the Big Five personality traits of conscientiousness, openness to experience, and lower neuroticism (Skowron & Dendy, 2004), as well as greater behavioral regulation and metacognitive ability (Bridgett et al., 2012). It has also been shown to be negatively correlated with emotional reactivity and avoidance (*r* = .42 - .50; (Skowron & Dendy, 2004), supporting convergent validity.

Exploratory Factor Analysis. First, we used a principal components analysis and found six Eigenvalues above 1 that explained 54.54% of the variance in the items. This may be accounted for by the reverse-coded items from each subscale loading on to their own factor. Next, we used principal axis factoring with varimax rotation and extracted three factors. The solution explained only 25.94% of variance and the items did not load on to the expected factors. The loadings can be found in Table 1.

| **Table 1.** *Factor Loadings of Effortful Control with Three Factors* | | | |
| --- | --- | --- | --- |
|  | 1 | 2 | 3 |
| EC2R. | .05 | .11 | .46 |
| EC5R | .10 | .28 | .41 |
| EC8R | .10 | .16 | .43 |
| EC11 | .44 | .15 | -.03 |
| EC15 | .64 | -.01 | -.00 |
| EC26 | .30 | .14 | .12 |
| EC27 | .62 | -.00 | .18 |
| EC29R | .31 | .45 | .37 |
| EC35 | .51 | .16 | .24 |
| EC40R | .09 | .43 | .15 |
| EC43 | .33 | .06 | .11 |
| EC47 | .33 | -.07 | .54 |
| EC50R | .20 | .54 | -.00 |
| EC53R | .05 | .45 | .13 |
| EC55 | .34 | -.09 | .41 |
| EC60R | .10 | .56 | .01 |
| EC63R | -.03 | .38 | .11 |
| EC72R | -.00 | .29 | .28 |
| EC76 | -.02 | -.13 | -.19 |

Next, we decided to examine a one-factors solution, to represent overall effortful control. This explained only 15.42% of variance in the items. The factor loadings can be found in Table 2. Neither solution was adequate, which accounts for the low reliability of the scale.

| **Table 2.** *Factor Loadings of Effortful Control with One Factor* | | |
| --- | --- | --- |
|  | 1 |  |
| EC2R | .34 |  |
| EC5R | .44 |  |
| EC8R | .38 |  |
| EC11 | .34 |  |
| EC15 | .37 |  |
| EC26 | .34 |  |
| EC27 | .47 |  |
| EC29R | .66 |  |
| EC35 | .55 |  |
| EC40R | .37 |  |
| EC43 | .30 |  |
| EC47 | .46 |  |
| EC50R | .39 |  |
| EC53R | .33 |  |
| EC55 | .39 |  |
| EC60R | .35 |  |
| EC63R | .23 |  |
| EC72R | .31 |  |
| EC76 | -.19 |  |

Confirmatory Factor Analysis. We conducted a confirmatory factor analysis with the lavaan package for R. The model fit for the three-factor solution was poor (χ^2^(149) = 542.14, *p* < .001; CFI = .69, RMSEA = .08, SRMR = .07). However, all but one item (EC76; “It is easy for me to inhibit fun behavior that would be inappropriate.”) loaded at above .2 on to the designated factors.

We also conducted a confirmatory factor analysis with just one factor—total effortful control. The fit for this model was poor (χ^2^(152) = 624.94, *p* < .001; CFI = .63, RMSEA = .09, SRMR = .08). Again, all but the same item loaded on to the factor at above .20.

Additional Validity Evidence.

*Five Facet Mindfulness Questionnaire (FFMQ; Baer et al., 2006)*

The FFMQ has been a standard in mindfulness research since its publication. It has high internal consistency (subscales α = .75 – 93), good convergent and discriminant validity, and has been reassessed using various diverse college student and community samples ((Baer et al., 2006; Christopher et al., 2012; Shallcross et al., 2020).

Exploratory Factor Analysis. First, we used a principal components analysis and found eight Eigenvalues above 1 that explained 57.57% of the variance in the items. Next, we used principal axis factoring with varimax rotation and extracted five factors. The solution explained only 41.39% of the variance but the items loaded on to the expected factors. The loadings can be found in Table 3.

| **Table 3.** *Factor Loadings of the FFMQ with Five Factors* | | | | | |
| --- | --- | --- | --- | --- | --- |
|  | Nonjudging | Acting w/ Awareness | Describing | Observing | Nonreacting |
| FFMQ_1 | -.11 | .04 | .02 | .44 | .17 |
| FFMQ_2 | .07 | .11 | .76 | .13 | .06 |
| FFMQ_3R | .57 | .19 | .02 | -.12 | .10 |
| FFMQ_4 | -.04 | .03 | .20 | .20 | .30 |
| FFMQ_5R | .12 | .77 | .09 | -.14 | .09 |
| FFMQ_6 | -.02 | .02 | .03 | .47 | .22 |
| FFMQ_7 | .16 | .02 | .63 | .24 | .18 |
| FFMQ_8R | .19 | .70 | .10 | -.05 | .03 |
| FFMQ_9 | .12 | .14 | .21 | .13 | .35 |
| FFMQ_10R | .59 | .04 | .11 | -.09 | .10 |
| FFMQ_11 | -.22 | .06 | .16 | .41 | .09 |
| FFMQ_12R | .28 | .21 | .67 | -.05 | -.05 |
| FFMQ_13R | .14 | .77 | .15 | -.13 | .07 |
| FFMQ_14R | .67 | .22 | .11 | -.13 | .10 |
| FFMQ_15 | -.05 | -.08 | .01 | .65 | .14 |
| FFMQ_16R | .31 | .27 | .67 | -.09 | -.03 |
| FFMQ_17R | .59 | .12 | .05 | -.16 | -.08 |
| FFMQ_18R | .32 | .56 | .13 | -.07 | -.01 |
| FFMQ_19 | -.03 | .02 | .09 | .26 | .56 |
| FFMQ_20 | -.03 | -.17 | -.01 | .58 | .13 |
| FFMQ_21 | .05 | -.03 | .18 | .32 | .42 |
| FFMQ_22R | .37 | .27 | .43 | -.04 | -.18 |
| FFMQ_23R | .41 | .36 | .08 | -.01 | -.16 |
| FFMQ_24 | -.03 | -.05 | .03 | .11 | .54 |
| FFMQ_25R | .76 | .14 | .14 | -.15 | -.01 |
| FFMQ_26 | -.14 | -.04 | .13 | .41 | .03 |
| FFMQ_27 | .06 | .05 | .62 | .18 | .35 |
| FFMQ_28R | .29 | .44 | .02 | .08 | -.13 |
| FFMQ_29 | -.01 | -.14 | .08 | .13 | .55 |
| FFMQ_30R | .77 | .17 | .08 | -.08 | .00 |
| FFMQ_31 | -.06 | -.11 | .11 | .55 | .09 |
| FFMQ_32 | -.02 | .01 | .43 | .31 | .24 |
| FFMQ_33 | .07 | -.05 | .00 | .14 | .52 |
| FFMQ_34R | .29 | .39 | .11 | .08 | -.21 |
| FFMQ_35R | .64 | .18 | .12 | -.07 | -.01 |
| FFMQ_36 | -.06 | .09 | .24 | .45 | .23 |
| FFMQ_37 | .03 | .05 | .59 | .30 | .24 |
| FFMQ_38R | .34 | .58 | .09 | .02 | -.14 |
| FFMQ_39R | .56 | .25 | .08 | .04 | .02 |
|  | | | | | |
|  | | | | | |

Confirmatory Factor Analysis. The model fit for the five-factor solution was adequate (χ^2^(692) = 2134.86, *p* < .001; CFI = .79, RMSEA = .07, SRMR = .09). All items loaded at above .4 onto their designated factors. We also conducted a confirmatory factor analysis with just one factor—total mindfulness. The fit for this model was poor (χ^2^(702) = 4721.45, *p* < .001; CFI = .42, RMSEA = .11, SRMR = .14).

*Considerations of Future Consequences Scale (CFC; Joireman et al., 2012)*

The updated 14 question scale was used in this study because unlike the original, it has clear support for a two-factor solution (Dassen et al., 2015; Joireman & Liu, 2014; Khachatryan et al., 2013). It has been tested within both student and community samples, and has an adequate internal consistency (Future: α = .80, Immediate: α = .84; (Joireman et al., 2012).

The original CFC, when used as a single factor indicating concern for the future, had a small but significant correlation with choosing immediate rewards on the Delay Discounting Task (Acuff et al., 2017) and Money Choice Questionnaire (Daugherty & Brase, 2010) in college student samples. It also had medium-large associations with the future (*r* = 0.44, *p* < .01) and present fatalistic perspectives (*r* = -0.46, *p* < .01) of the Zimbardo Time Perspective Inventory (Daugherty & Brase, 2010).

Exploratory Factor Analysis. First, we used a principal components analysis and found three Eigenvalues above 1 that explained 54.94% of the variance in the items. Next, we used principal axis factoring with varimax rotation and extracted two factors. The solution explained only 38.73% of the variance but the items loaded on to the expected factors. The loadings can be found in Table 4.

| **Table 4.** *Factor Loadings for Considerations of Future Consequences Scale with Two Factors* | | | |
| --- | --- | --- | --- |
|  | Immediate | Future |  |
| CFC_1 | -.02 | .68 |  |
| CFC_2 | -.02 | .55 |  |
| CFC_3 | .67 | -.04 |  |
| CFC_4 | .64 | -.03 |  |
| CFC_5 | .45 | .22 |  |
| CFC_6 | .04 | .58 |  |
| CFC_7 | -.07 | .62 |  |
| CFC_8 | .19 | .36 |  |
| CFC_9 | .56 | -.12 |  |
| CFC_10 | .63 | -.17 |  |
| CFC_11 | .72 | -.09 |  |
| CFC_12 | .67 | .06 |  |
| CFC_13 | -.18 | .67 |  |
| CFC_14 | -.13 | .64 |  |

Confirmatory Factor Analysis. The model fit for the two-factor solution was adequate (χ^2^(76) = 379.15, *p* < .001; CFI = .84, RMSEA = .09, SRMR = .08). All items loaded onto their designated factor at above .3.

Study 2

*Adult Temperament Questionnaire-Effortful Control (ATQ-SF; Evans & Rothbart, 2007)*

Exploratory Factor Analysis. First, we used a principal components analysis and found five Eigenvalues above 1 that explained 52.89% of the variance in the items. Next, we used principal axis factoring with varimax rotation and extracted three factors. The solution explained only 41.06% of variance and the items did not load on to the expected factors. However, the loadings were more accurate than in Study 1. The loadings can be found in Table 5.

| **Table 5.** *Factor Loadings of Effortful Control with Three Factors* | | | |
| --- | --- | --- | --- |
|  | 1 | 2 | 3 |
| EC2R | .07 | .04 | .75 |
| EC5R | .26 | .05 | .35 |
| EC8R | .20 | .01 | .73 |
| EC11 | .34 | .48 | .04 |
| EC15 | .10 | .76 | .10 |
| EC26 | .34 | .22 | .01 |
| EC27 | .03 | .78 | .11 |
| EC29R | .48 | .40 | .35 |
| EC35 | .35 | .57 | .19 |
| EC40R | .43 | .18 | .16 |
| EC43 | .40 | .44 | -.09 |
| EC47 | -.03 | .56 | .49 |
| EC50R | .74 | .11 | .11 |
| EC53R | .45 | .17 | -.02 |
| EC55 | -.12 | .40 | .61 |
| EC60R | .68 | -.07 | .15 |
| EC63R | .60 | .04 | .08 |
| EC72R | .33 | .08 | .35 |
| EC76 | .22 | .23 | -.21 |

Confirmatory Factor Analysis. We conducted a confirmatory factor analysis with the lavaan package for R. The model fit for the three-factor solution was poor (χ^2^(149) = 490.64, *p* < .001; CFI = .76, RMSEA = .08, SRMR = .07). However, all but one item (EC76; “It is easy for me to inhibit fun behavior that would be inappropriate.”) loaded at above .30 on to the designated factors.

We also conducted a confirmatory factor analysis with just one factor—total effortful control. The fit for this model was poor (χ^2^(152) = 605.13, *p* < .001; CFI = .68, RMSEA = .09, SRMR = .08). Again, all but the same item loaded on to the factor at above .30.

*Five Facet Mindfulness Questionnaire (FFMQ; Baer et al., 2006)*

Exploratory Factor Analysis. First, we used a principal components analysis and found nine Eigenvalues above 1 that explained 60.86% of the variance in the items. Next, we used principal axis factoring with varimax rotation and extracted five factors. The solution explained only 42.54% of the variance, but the items loaded on to the expected factors. The loadings can be found in Table 6.

| **Table 6.** *Factor Loadings of Five Facet Mindfulness Questionnaire with Five Factors* | | | | | |
| --- | --- | --- | --- | --- | --- |
|  | Factor | | | | |
|  | Nonjudging | Describing | Acting w/ Awareness | Observing | Nonreacting |
| FFMQ_1 | -.17 | -.06 | -.02 | .49 | .06 |
| FFMQ_2 | .08 | .74 | .01 | .17 | .04 |
| FFMQ_3R | .67 | .10 | .06 | -.07 | .05 |
| FFMQ_4 | -.01 | .17 | .14 | .09 | .35 |
| FFMQ_5R | .06 | .00 | .70 | -.05 | .19 |
| FFMQ_6 | -.01 | -.01 | .05 | .44 | .05 |
| FFMQ_7 | .11 | .68 | .10 | .06 | .11 |
| FFMQ_8R | .10 | .06 | .67 | -.02 | .17 |
| FFMQ_9 | .02 | .18 | .17 | .13 | .52 |
| FFMQ_10R | .68 | .11 | .14 | -.07 | .02 |
| FFMQ_11 | -.04 | .05 | .01 | .49 | -.01 |
| FFMQ_12R | .16 | .69 | .15 | -.20 | .07 |
| FFMQ_13R | .07 | .03 | .74 | -.04 | .23 |
| FFMQ_14R | .79 | .09 | .17 | -.06 | .05 |
| FFMQ_15 | -.07 | -.01 | -.08 | .56 | .08 |
| FFMQ_16R | .16 | .76 | .21 | -.19 | .07 |
| FFMQ_17R | .54 | .01 | .06 | -.19 | -.11 |
| FFMQ_18R | .13 | .16 | .60 | -.05 | .31 |
| FFMQ_19 | .00 | .17 | .15 | .31 | .39 |
| FFMQ_20 | -.15 | -.09 | -.06 | .63 | .05 |
| FFMQ_21 | .17 | .05 | .09 | .27 | .39 |
| FFMQ_22R | .21 | .52 | .19 | -.08 | .10 |
| FFMQ_23R | .26 | .17 | .49 | .02 | .08 |
| FFMQ_24 | -.02 | .06 | .07 | .01 | .46 |
| FFMQ_25R | .77 | .11 | .16 | -.15 | .10 |
| FFMQ_26 | -.07 | .13 | .07 | .58 | .02 |
| FFMQ_27 | .11 | .44 | .02 | .14 | .18 |
| FFMQ_28R | .18 | .17 | .55 | .08 | .05 |
| FFMQ_29 | .00 | .10 | .05 | .06 | .69 |
| FFMQ_30R | .79 | .13 | .14 | -.05 | .03 |
| FFMQ_31 | -.04 | .09 | .02 | .56 | .17 |
| FFMQ_32 | -.06 | .52 | .06 | .28 | .11 |
| FFMQ_33 | .07 | .01 | .07 | .02 | .51 |
| FFMQ_34R | .16 | .11 | .60 | .11 | -.08 |
| FFMQ_35R | .72 | .12 | .11 | -.09 | .07 |
| FFMQ_36 | -.06 | .30 | .01 | .50 | .06 |
| FFMQ_37 | .10 | .74 | .07 | .16 | .11 |
| FFMQ_38R | .15 | .07 | .71 | .02 | .02 |
| FFMQ_39R | .60 | .08 | .18 | -.04 | .02 |

Confirmatory Factor Analysis. The model fit for the five-factor solution was adequate (χ^2^(692) = 1555.25, *p* < .001; CFI = .84, RMSEA = .06, SRMR = .07). All items loaded at above .4 onto their designated factors. We also conducted a confirmatory factor analysis with just one factor—total mindfulness. The fit for this model was poor (χ^2^(702) = 4092.94, *p* < .001; CFI = .38, RMSEA = .11, SRMR = .13).

*Considerations of Future Consequences Scale (CFC; Joireman et al., 2012)*

Exploratory Factor Analysis. First, we used a principal components analysis and found two Eigenvalues above 1 that explained 54.29% of the variance in the items. Next, we used principal axis factoring with varimax rotation and extracted two factors. The solution explained only 47.14% of the variance but the items loaded on to the expected factors. The loadings can be found in Table 7.

| **Table 7.** *Factor Loadings of Considerations of Future Consequences Scale* | | |
| --- | --- | --- |
|  | Immediate | Future |
| CFC_1 | -.21 | .67 |
| CFC_2 | -.28 | .58 |
| CFC_3 | .77 | -.14 |
| CFC_4 | .76 | -.14 |
| CFC_5 | .63 | -.02 |
| CFC_6 | -.31 | .53 |
| CFC_7 | -.23 | .57 |
| CFC_8 | .04 | .38 |
| CFC_9 | .57 | -.29 |
| CFC_10 | .62 | -.29 |
| CFC_11 | .76 | -.30 |
| CFC_12 | .64 | -.17 |
| CFC_13 | -.19 | .75 |
| CFC_14 | -.17 | .77 |

Confirmatory Factor Analysis. The model fit for the two-factor solution was adequate (χ^2^(76) = 328.89, *p* < .001; CFI = .89, RMSEA = .09, SRMR = .06). All items loaded onto their designated factor at above .30.

## Additional Tables—Studies 1 and 2

**Table 8.** *Descriptive Statistics for Continuous Media Multitasking Variables in Studies 1 and 2*

|  | Study 1 (*N =* 487) | | | | Study 2 (*N* = 381) | | | |
| --- | --- | --- | --- | --- | --- | --- | --- | --- |
|  | *M* | *SD* | Range | α | *M* | *SD* | Range | α |
| Overall Hours | 33.80 | 21.00 | 0.00 – 240.00 |  | 23.03 | 11.37 | 5.20 - 92.00 |  |
| Overall Media Multitasking | 3.06 | 1.44 | 0.12-9.71 | .64 | 2.68 | 1.21 | .42 - 8.15 | .55 |
| MMI Face-to-Face | 0.53 | 0.43 | 0.00 - 2.39 | .89 | 0.53 | 0.46 | 0.00 - 5.22 | .87 |
| MMI Print | 0.09 | 0.16 | 0.00 - 1.50 | .89 | 0.07 | 0.11 | 0.00 - 0.91 | .89 |
| MMI Texting | 0.44 | 0.36 | 0.00 - 2.09 | .83 | 0.30 | 0.28 | 0.00 - 1.89 | .80 |
| MMI Social Media | 0.46 | 0.34 | 0.00 - 2.22 | .84 | 0.37 | 0.30 | 0.00 - 1.54 | .81 |
| MMI Non-Social | 0.10 | 0.16 | 0.00 - 1.29 | .88 | 0.07 | 0.11 | 0.00 - 0.61 | .83 |
| MMI Phone or Video Chat | 0.20 | 0.22 | 0.00 - 1.60 | .84 | 0.09 | 0.14 | 0.00 - 1.18 | .85 |
| MMI Audio | 0.44 | 0.39 | 0.00 - 3.07 | .80 | 0.40 | 0.38 | 0.00 - 2.52 | .79 |
| MMI Video | 0.28 | 0.27 | 0.00 - 1.73 | .85 | 0.27 | 0.24 | 0.00 - 1.25 | .86 |
| MMI Games | 0.13 | 0.22 | 0.00 - 1.47 | .88 | 0.10 | 0.16 | 0.00 - 0.90 | .87 |
| MMI Homework | 0.39 | 0.29 | 0.00 - 2.10 | .81 | 0.47 | 0.30 | 0.00 - 1.63 | .81 |
| Texting in BG | 43.47 | 20.24 | 0.50 - 100.00 | .89 | 39.43 | 17.56 | 6.25 - 100.00 | .91 |
| Social Media in BG | 39.65 | 20.14 | 0.00 - 99.10 | .88 | 40.39 | 18.56 | 4.25 - 99.80 | .91 |
| Phone in BG | 29.67 | 20.78 | 0.00 - 96.70 | .89 | 27.94 | 16.23 | 1.80 - 90.00 | .89 |
| Audio in BG | 40.76 | 21.28 | 0.00 - 100.00 | .87 | 47.13 | 18.43 | 4.00 - 98.75 | .86 |
| Video in BG | 28.03 | 19.30 | 0.00 - 99.00 | .89 | 33.92 | 16.91 | 2.00 - 95.25 | .89 |

*Note*. MMI = Media Multitasking Index. BG = background. α = Cronbach’s α.

| **Table 9.** *Descriptive Statistics for Survey Measures in Studies 1 and 2* | | | | | | | | | |
| --- | --- | --- | --- | --- | --- | --- | --- | --- | --- |
|  | Study 1 (*N* = 487) | | | | Study 2 (*N* = 381) | | | |  |
|  | *M* | *SD* | Range | α | *M* | *SD* | Range | α |  |
| FFMQ Total | 119.6 | 16.1 | 65.0 – 182.0 | .86 | 122.2 | 15.7 | 89.0 – 172.0 | .87 |  |
| FFMQ Observing | 26.5 | 5.0 | 11.0 – 40.0 | .76 | 26.8 | 5.0 | 9.0 – 40.0 | .77 |  |
| FFMQ Describing | 24.6 | 5.8 | 8.0 – 40.0 | .85 | 26.0 | 5.5 | 11.0 – 40.0 | .86 |  |
| FFMQ Acting with Awareness | 24.1 | 5.7 | 9.0 – 39.0 | .85 | 23.6 | 5.6 | 9.0 – 40.0 | .86 |  |
| FFMQ Nonjudging | 23.7 | 6.4 | 8.0 – 40.0 | .87 | 24.7 | 6.6 | 8.0 – 39.0 | .89 |  |
| FFMQ Nonreactivity | 20.7 | 4.0 | 7.0 – 33.0 | .72 | 21.2 | 3.9 | 9.0 – 33.0 | .71 |  |
| ATQ EC | 78.6 | 12.4 | 39.0 – 118.0 | .74 | 79.2 | 15.0 | 38.0 – 125.0 | .81 |  |
| ATQ Activation Control | 31.5 | 6.2 | 11.0 – 48.0 | .61 | 32.7 | 7.0 | 12.0 – 49.0 | .72 |  |
| ATQ Attentional Control | 18.5 | 4.8 | 5.0 – 33.0 | .65 | 17.6 | 5.0 | 7.0 – 32.0 | .68 |  |
| ATQ Inhibitory Control | 28.6 | 5.5 | 12.0 – 45.0 | .42 | 29.0 | 6.6 | 12.0 – 47.0 | .60 |  |
| CFC Future | 32.4 | 6.8 | 0.0 – 49.0 | .78 | 33.6 | 6.9 | 13.0 – 49.0 | .83 |  |
| CFC Immediate | 26.4 | 7.1 | 0.0 – 49.0 | .81 | 23.9 | 7.6 | 7.0 – 49.0 | .87 |  |
| MCQ log *k* |  |  |  |  | -2.20 | .60 | -3.12 – -.88 |  |  |
|  |  |  |  |  |  |  |  |  |  |

*Note.* FFMQ = Five Facet Mindfulness Questionnaire, ATQ = Adult Temperament Questionnaire, EC = Effortful Control, CFC = Considerations of Future Consequences Scale, MCQ = Money Choice Questionnaire. α = Cronbach’s α.

| **Table 10.** *Behavioral Measures Characteristics in Studies 1 and 2* | | | | | | |
| --- | --- | --- | --- | --- | --- | --- |
|  | Study 1 (*N* = 487^a^) | | | Study 2 (*N* = 381) | | |
| Variable | *M* | *SD* | Range | *M* | *SD* | Range |
| Survey Time Estimation (Midway) | 0.94 | 0.58 | 0.00 – 4.57 | 1.04 | 0.51 | 0.31 – 9.50 |
| Survey Time Estimation (End) | 0.86 | 0.48 | 0.01 – 3.06 | 1.02 | 0.27 | 0.16 – 2.53 |
| Survey Time Estimation Average | 0.91 | 0.52 | 0.01 – 3.09 | 1.03 | 0.35 | 0.25 – 5.75 |
| log Estimation Avg |  |  |  | -.00 | .12 | -.59 - .76 |
| Time Production Task (5s and 8s) | 0.92 | 0.24 | 0.05 – 1.74 |  |  |  |
| Time Production Task (18s and 23s) | 0.81 | 0.24 | 0.01 – 1.56 |  |  |  |
| Time Production Task Average | 0.86 | 0.22 | 0.03 – 1.60 |  |  |  |
| Stop Signal Go Arrow Reaction Time | 342.41 | 31.38 | 129.94 – 423.77 | 339.44 | 24.14 | 283.18 – 414.32 |
| Stop Signal Omission Error | 2.48 | 4.98 | 0.00 – 40.00 | 1.22 | 1.81 | 0.00 – 18.00 |
| sqrt Omission |  |  |  | 4.24 | 0.80 | 0.00 – 4.24 |
| Stop Signal Commission Error | 3.35 | 3.24 | 0.00 – 22.00 | 2.56 | 2.38 | 0.00 – 26.00 |
| sqrt Commission |  |  |  | 1.42 | 0.76 | 0.00 – 5.10 |

*Note*. ^a^ The sample size for the Time Production Task and Stop Signal Task in Study 1 is 300.

## Additional Tables—Study 1

| 16 |  |  |  |  |  |  |  |  |  |  |  |  |  |  |  | 1 |
| --- | --- | --- | --- | --- | --- | --- | --- | --- | --- | --- | --- | --- | --- | --- | --- | --- |
| 15 |  |  |  |  |  |  |  |  |  |  |  |  |  |  | 1 | .22^**^ |
| 14 |  |  |  |  |  |  |  |  |  |  |  |  |  | 1 | -.16^**^ | -.19^**^ |
| 13 |  |  |  |  |  |  |  |  |  |  |  |  | 1 | -.02 | -.06 | -.10 |
| 12 |  |  |  |  |  |  |  |  |  |  |  | 1 | .35^**^ | 0.02 | -.03 | -.10 |
| 11 |  |  |  |  |  |  |  |  |  |  | 1 | .42^**^ | .29^**^ | -.01 | -.02 | -.10 |
| 10 |  |  |  |  |  |  |  |  |  | 1 | .79^**^ | .75^**^ | .72^**^ | -.01 | -.05 | -.13^*^ |
| 9 |  |  |  | *Note.* MMI = Media Multitasking Index, MM = Real-time in-study Media Multitasking during survey and E-Prime tasks, FFMQ = Five Facet Mindfulness Questionnaire, ATQ = Adult Temperament Questionnaire, EC = Effortful Control, SST = Stop Signal Task, RT = Reaction Time (ms). Omission errors were indicated by misses on “go” arrows and commission errors were indicated by key presses on “stop” arrows. Two-tailed: * *p* < .05. ** *p* < .01.  Numbers in bold are those that pertain to the study hypotheses. |  |  |  |  | 1 | .24^**^ | .18^**^ | .16^**^ | .20^**^ | -.02 | -.05 | -.19^**^ |
| 8 |  |  |  |  |  |  |  | 1 | .02 | .33^**^ | .25^**^ | .35^**^ | .16^**^ | .14^**^ | .08 | -.04 |
| 7 | **Table 11.** *Summary of Zero-Order Correlation Analysis on Cognitive Control and Media Multitasking Variables in Study 1 (N = 297 – 487)* |  |  |  |  |  | 1 | .53^**^ | -.04 | .57^**^ | .40^**^ | .61^**^ | .32^**^ | .14^**^ | .01 | -.11 |
| 6 |  |  |  |  |  | 1 | .34^***^ | .33^***^ | .31^***^ | .37^***^ | .32^***^ | .33^***^ | .20^***^ | .11 | .11 | -.17^**^ |
| 5 |  |  |  |  | 1 | .25^***^ | -.13^**^ | -.21^***^ | .43^***^ | .02 | .07 | -.04 | -.01 | -.01 | -.01 | -.02 |
| 4 |  |  |  | 1 | .38^***^ | .77^***^ | .64^***^ | .65^***^ | .49^***^ | .53^***^ | .42^***^ | .50^***^ | .29^***^ | .13^*^ | .06 | -.17^**^ |
| 3 |  |  | 1 | -.06 | -.01 | -.06 | -.08 | -.04 | .03 | -.12^*^ | -.07 | -.09 | -.12^*^ | .00 | .14^**^ | .05 |
| 2 |  | 1 | .13^*^ | -.08^*^ | .03 | -.10^*^ | -.07 | -.10^*^ | .06 | -.13^**^ | -.16^***^ | -.11^**^ | -.02 | -.01 | -.04 | -.02 |
| 1 | 1 | .01 | .17^**^ | **-.02** | **.09** | **-.02** | **-.06** | **-.10^*^** | **.08^*^** | **-.05** | **-.01** | **.02** | **-.11^**^** | -.01 | **.08** | **.09** |
| Variables | 1. MMI | 2. MMT Survey | 3. MMT E-Prime | 4. FFMQ | 5. FFMQ Observing | 6. FFMQ Describing | 7. FFMQ Act Aware | 8. FFMQ Nonjudge | 9. FFMQ Nonreact | 10. ATQ EC | 11. ATQ EC Activation | 12. ATQ EC Attentional | 13. ATQ EC Inhibitory | 14. SST Go RT | 15.sqrt SST Omission | 16. sqrt SST Commission |

**Table 12.** *Summary of Correlation Analysis on Cognitive Control and Media Multitasking Variables, Controlling for Age, Gender, Ethnicity, Whether Participants Used Screen Time App, and Whether They Participated in E-Prime Portion in Study 1 (N = 471)*

| 12 |  |  |  |  |  |  |  |  |  |  |  | 1 |
| --- | --- | --- | --- | --- | --- | --- | --- | --- | --- | --- | --- | --- |
| 11 |  |  |  |  |  |  |  |  |  |  | 1 | .34^**^ |
| 10 |  |  |  |  |  |  |  |  |  | 1 | .41^**^ | .28^**^ |
| 9 |  |  |  |  |  |  |  |  | 1 | .78^**^ | .75^**^ | .72^**^ |
| 8 |  |  |  |  |  |  |  | 1 | .20^**^ | .15^**^ | .12^**^ | .18^**^ |
| 7 |  |  |  |  |  |  | 1 | .02 | .32^**^ | .24^**^ | .35^**^ | .16^**^ |
| 6 |  |  |  |  |  | 1 | .35^**^ | -.07 | .57^**^ | .39^**^ | .61^**^ | .32^**^ |
| 5 |  |  |  |  | 1 | .34^**^ | .34^**^ | .29^**^ | .36^**^ | .31^**^ | .32^**^ | .19^**^ |
| 4 |  |  |  | 1 | .26^**^ | -.13^**^ | -.21^**^ | .44^**^ | .02 | .06 | -.03 | .00 |
| 3 |  |  | 1 | .38^**^ | .77^**^ | .63^**^ | .65^**^ | .47^**^ | .52^**^ | .40^**^ | .49^**^ | .29^**^ |
| 2 |  | 1 | -.07 | .04 | -.10^*^ | -.08 | -.10^*^ | .07 | -.14^**^ | -.16^**^ | -.12^*^ | -.03 |
| 1 | 1 | .02 | **-.02** | **.08** | **-.01** | **-.06** | **-.11^*^** | **.09** | **-.04** | **-.01** | **.03** | **-.10^*^** |
| Variables | 1. MMI | 2. MMT Survey | 3. FFMQ | 4. FFMQ Observing | 5. FFMQ Describing | 6. FFMQ Act Aware | 7. FFMQ Nonjudge | 8. FFMQ Nonreact | 9. ATQ Effortful Control | 10. ATQ EC Activation | 11. ATQ EC Attentional | 12. ATQ EC Inhibitory |

*Note.* MMI = Media Multitasking Index, MM = Real-time in-study Media Multitasking during survey and E-Prime tasks, FFMQ = Five Facet Mindfulness Questionnaire, ATQ = Adult Temperament Questionnaire, EC = Effortful Control. Numbers in bold are those that pertain to the study hypotheses. Two-tailed: * *p* < .05. ** *p* < .01.

**Table 13.** *Summary of Correlation Analysis on Cognitive Control and Media Multitasking Variables, Controlling for Age, Gender, Ethnicity, and Whether Participants Used Screen Time App in Study 1, in Subsample that Completed Both Parts of Study (N = 286)*

| 16 |  |  |  |  |  |  |  |  |  |  |  |  |  |  |  | 1 |
| --- | --- | --- | --- | --- | --- | --- | --- | --- | --- | --- | --- | --- | --- | --- | --- | --- |
| 15 |  |  |  |  |  |  |  |  |  |  |  |  |  |  | 1 | .29^***^ |
| 14 |  |  |  |  |  |  |  |  |  |  |  |  |  | 1 | 0.13^*^ | -.21^***^ |
| 13 |  |  |  |  |  |  |  |  |  |  |  |  | 1 | .03 | .01 | -.09 |
| 12 |  |  |  |  |  |  |  |  |  |  |  | 1 | .37^**^ | .06 | .00 | -.08 |
| 11 |  |  |  |  |  |  |  |  |  |  | 1 | .43^**^ | .31^**^ | .00 | .03 | -.10 |
| 10 |  |  |  |  |  |  |  |  |  | 1 | .80^**^ | .75^**^ | .72^**^ | .03 | -.02 | -.12^*^ |
| 9 |  |  |  |  |  |  |  |  | 1 | .21^**^ | .18^**^ | .11 | .17^**^ | .02 | -.03 | -.18^**^ |
| 8 |  |  |  |  |  |  |  | 1 | .06 | .31^**^ | .21^**^ | .35^**^ | .17^**^ | .14^*^ | .04 | -.02 |
| 7 |  |  |  |  |  |  | 1 | .51^**^ | -.02 | .57^**^ | .39^**^ | .62^**^ | .33^**^ | .15^*^ | .03 | -.10 |
| 6 |  |  |  |  |  | 1 | .32^**^ | .31^**^ | .35^**^ | .39^**^ | .35^**^ | .35^**^ | .18^**^ | .15^*^ | .07 | -.15^**^ |
| 5 |  |  |  |  | 1 | .29^**^ | -.05 | -.20^**^ | .44^**^ | .05 | .08 | -.01 | .02 | -.03 | -.02 | -.04 |
| 4 |  |  |  | 1 | .42^**^ | .76^**^ | .64^**^ | .64^**^ | .52^**^ | .53^**^ | .41^**^ | .51^**^ | .29^**^ | .16^*^ | .04 | -.15^**^ |
| 3 |  |  | 1 | -.08 | -.01 | -.08 | -.09 | -.06 | .03 | -.12^*^ | -.07 | -.10 | -.11 | .00 | -.05 | .07 |
| 2 |  | 1 | .14^*^ | -.12 | .03 | -.12* | -.11 | -.13* | .07 | -.18^**^ | -.16^**^ | -.17^**^ | -.09 | .01 | -.02 | -.01 |
| 1 | 1 | .04 | .17^**^ | **-.04** | **.11** | **-.06** | **-.09** | **-.09** | **.07** | **-.06** | **-.03** | **.03** | **-.12** | -.04 | **.08** | **.09** |
| Variables | 1. MMI | 2. MMT Survey | 3. MMT E-Prime | 4. FFMQ | 5. FFMQ Observing | 6. FFMQ Describing | 7. FFMQ Act Aware | 8. FFMQ Nonjudge | 9. FFMQ Nonreact | 10. ATQ EC | 11. ATQ EC Activation | 12. ATQ EC Attentional | 13. ATQ EC Inhibitory | 14. SST Go RT | 15. sqrt SST Omission | 16. sqrt SST Commission |

*Note.* MMI = Media Multitasking Index, MM = Real-time in-study Media Multitasking during survey and E-Prime tasks, FFMQ = Five Facet Mindfulness Questionnaire, ATQ = Adult Temperament Questionnaire, EC = Effortful Control, SST = Stop Signal Task, RT = Reaction Time (ms). Omission errors were indicated by misses on “go” arrows and commission errors were indicated by key presses on “stop” arrows. Numbers in bold are those that pertain to the study hypotheses. Two-tailed: * *p* < .05. ** *p* < .01.

**Table 14.** *Summary of Zero-Order Correlation Analysis on Time Perspective and Media Multitasking Variables in Study 1 (N = 487^a^)*

| Variables | 1 | 2 | 3 | 4 | 5 | 6 | 7 | 8 | 9 | 10 | 11 | |
| --- | --- | --- | --- | --- | --- | --- | --- | --- | --- | --- | --- | --- |
| 1. MMI | 1 |  |  |  |  |  |  |  |  |  |  | |
| 2. MMT Survey | .01 | 1 |  |  |  |  |  |  |  |  |  | |
| 3. MMT E-Prime | .17^**^ | .13^*^ | 1 |  |  |  |  |  |  |  |  | |
| 4. STE (Mid) | .04 | **-.13^**^** | .03 | 1 |  |  |  |  |  |  |  | |
| 5. STE (End) | .01 | **-.16^***^** | -.01 | .75^***^ | 1 |  |  |  |  |  |  | |
| 6. STE (Avg) | .03 | **-.15^**^** | .02 | .95^***^ | .92^***^ | 1 |  |  |  |  |  | |
| 7. TPT (5, 8s) | **-.03** | .04 | .06 | -.00 | .03 | .02 | 1 |  |  |  |  | |
| 8. TPT (18, 23s) | **-.10** | .05 | .02 | .00 | .01 | .01 | .81^***^ | 1 |  |  |  | |
| 9. TPT (Avg) | **-.07** | .05 | .04 | .00 | .02 | .01 | .95^***^ | .95^***^ | 1 |  |  | |
| 10. CFC Future | **.02** | -.09^*^ | .08 | -.03 | -.04 | -.04 | .09 | .10 | .10 | 1 |  | |
| 11. CFC Immediate | **.20^***^** | .06 | -.02 | .06 | .05 | .06 | .04 | .04 | .04 | -.03 | 1 | |
| *Note*. MMI = Media Multitasking Index, MMT = Media Multitasking, STE = Survey Time Estimation, TPT = Time Production Task, CFC = Considerations of Future Consequences scale. Numbers in bold are those that pertain to the study hypotheses.  ^a^ *N* = 297 for correlations with media multitasking during E-Prime portion and time production task variables.   \| Two-tailed significance: * *p* < .05. ** *p* < .01. *** *p* < .001. \| \| --- \| | | | | | | | | | | | |  |

**Table 15.** *Summary of Correlation Analysis on Time Perspective and Media Multitasking Variables, Controlling for Age, Gender, Ethnicity, and Whether Participants Used Screen Time App and Whether They Participated in E-Prime Portion in Study 1 (N = 468)*

| Variables | 1 | 2 | 3 | 4 | 5 | 6 | 7 |
| --- | --- | --- | --- | --- | --- | --- | --- |
| 1. MMI | 1 |  |  |  |  |  |  |
| 1. MMT Survey | .02 | 1 |  |  |  |  |  |
| 1. STE (Mid) | .05 | **-.12^*^** | 1 |  |  |  |  |
| 1. STE (End) | .02 | **-.14^**^** | .73^***^ | 1 |  |  |  |
| 1. STE (Avg) | .04 | **-.14^**^** | .94^***^ | .92^***^ | 1 |  |  |
| 1. CFC Future | **.01** | -.09 | -.03 | -.05 | -.04 | 1 |  |
| 1. CFC Immediate | **.21^***^** | .07 | .06 | .03 | .05 | -.11^*^ | 1 |
| *Note*. MMI = Media Multitasking Index, MMT = Media Multitasking, STE = Survey Time Estimation, CFC = Considerations of Future Consequences scale. Numbers in bold are those that pertain to the study hypotheses.   \| Two-tailed significance: * *p* < .05. ** *p* < .01. *** *p* < .001. \| \| --- \| | | | | | | | |

**Table 16.** *Summary of Correlation Analysis on Time Perspective and Media Multitasking Variables, Controlling for Age, Gender, Ethnicity, and Whether Participants Used Screen Time App in Study 1 (N = 287)*

| Variables | 1 | 2 | 3 | 4 | 5 | 6 | 7 | 8 | 9 | 10 | 11 | |
| --- | --- | --- | --- | --- | --- | --- | --- | --- | --- | --- | --- | --- |
| 1. MMI | 1 |  |  |  |  |  |  |  |  |  |  | |
| 1. MMT Survey | .05 | 1 |  |  |  |  |  |  |  |  |  | |
| 1. MMT E-Prime | .17^**^ | .13^*^ | 1 |  |  |  |  |  |  |  |  | |
| 1. STE (Mid) | .13^*^ | **-.13^*^** | .04 | 1 |  |  |  |  |  |  |  | |
| 1. STE (End) | .10 | **-.12*** | .00 | .66^***^ | 1 |  |  |  |  |  |  | |
| 1. STE (Avg) | .13 | **-.14^*^** | .02 | .94^***^ | .89^***^ | 1 |  |  |  |  |  | |
| 1. TPT (5, 8s) | **-.04** | .06 | .06 | -.01 | .02 | .00 | 1 |  |  |  |  | |
| 1. TPT (18, 23s) | **-.09** | .07 | .02 | -.02 | -.02 | -.02 | .81^***^ | 1 |  |  |  | |
| 1. TPT (Avg) | **-.07** | .07 | .04 | -.02 | .00 | -.01 | .95^***^ | .95^***^ | 1 |  |  | |
| 1. CFC Future | **.03** | -.14^*^ | .08 | -.06 | -.11 | -.09 | .06 | .06 | .06 | 1 |  | |
| 1. CFC Immediate | **.23^***^** | .13^*^ | -.01 | .06 | .06 | .07 | .03 | .03 | .03 | -.12^*^ | 1 | |
| *Note*. MMI = Media Multitasking Index, MMT = Media Multitasking, STE = Survey Time Estimation, TPT = Time Production Task, CFC = Considerations of Future Consequences scale. Numbers in bold are those that pertain to the study hypotheses.   \| Two-tailed significance: * *p* < .05. ** *p* < .01. *** *p* < .001. \| \| --- \| | | | | | | | | | | | |  |

## Additional Blockwise Regression Analyses—Study 1

The second blockwise regression analysis excluded Stop Signal and Time Production Task data but included a greater portion of the sample (*n* = 476; see Table 17). Here, having an immediate present-focused time perspective (β = .21, *p* < .001) and being younger (β = -.11, *p* = .019) significantly predicted media multitasking tendency. The final model was significant (*F*(10,466) = 3.05, *p* < .001) and described 4.1% (adjusted *R^2^*) of the variance in Media Multitasking Score. A sensitivity analysis indicated that a sample size of *N* = 464 at a power of 80%, with alpha level α = .05, with three tested indicators and eleven total indicators was large enough to detect an effect size of f^2^ = .02.

| **Table 17.** *Summary of Blockwise Multiple Regression Analysis of Survey-Derived Variables on Media Multitasking Index Score, in Total Sample (N = 476)* | | | | | | | | | | | | | |
| --- | --- | --- | --- | --- | --- | --- | --- | --- | --- | --- | --- | --- | --- |
| Model | |  | |  |  |  | Correlations | | | Collinearity Statistics | | |  |
|  |  | b | *SE* | β | *t* | *p* | Zero-order | Partial | Part | Tolerance | VIF |  |  |
| 1 | (Constant) | 8.89 | 2.39 |  | 3.73 | <.001 |  |  |  |  |  |  |  |
|  | Screen Time App | -0.07 | 0.13 | -.03 | -0.54 | .591 | -.02 | -.03 | -.02 | .99 | 1.02 |  |  |
|  | Gender | 0.16 | 0.13 | .06 | 1.20 | .231 | .06 | .06 | .05 | .98 | 1.02 |  |  |
|  | Age | -4.72 | 1.86 | -.12 | -2.54 | .011 | -.11 | -.12 | -.12 | 1.00 | 1.01 |  |  |
|  | Ethnicity | 0.08 | 0.05 | .07 | 1.57 | .116 | .06 | .07 | .07 | .99 | 1.01 |  |  |
|  | Whether they did E-Prime portion | -0.18 | 0.14 | -.06 | -1.35 | .179 | -.06 | -.06 | -.06 | .99 | 1.01 |  |  |
| 2 | (Constant) | 8.92 | 2.48 |  | 3.60 | <.001 |  |  |  |  |  |  |  |
|  | Screen Time App | -0.07 | 0.13 | -.03 | -0.54 | .587 | -.02 | -.03 | -.03 | .98 | 1.02 |  |  |
|  | Gender | 0.14 | 0.13 | .05 | 1.08 | .281 | .06 | .05 | .05 | .96 | 1.05 |  |  |
|  | Age | -4.71 | 1.86 | -.12 | -2.53 | .012 | -.11 | -.12 | -.12 | .99 | 1.01 |  |  |
|  | Ethnicity | 0.08 | 0.05 | .07 | 1.60 | .110 | .06 | .07 | .07 | .99 | 1.01 |  |  |
|  | Whether they did E-Prime portion | -0.17 | 0.14 | -.06 | -1.27 | .205 | -.06 | -.06 | -.06 | .99 | 1.02 |  |  |
|  | FFMQ | 0.15 | 0.19 | .04 | 0.78 | .435 | .02 | .04 | .04 | .72 | 1.40 |  |  |
|  | Effortful Control | -0.12 | 0.12 | -.05 | -0.99 | .323 | -.04 | -.05 | -.05 | .70 | 1.44 |  |  |
| 3 | (Constant) | 6.98 | 2.44 |  | 2.86 | .004 |  |  |  |  |  |  |  |
|  | Screen Time App | -0.03 | 0.13 | -.01 | -0.19 | .850 | -.02 | -.01 | -.01 | .98 | 1.03 |  |  |
|  | Gender | 0.15 | 0.13 | .05 | 1.17 | .241 | .06 | .05 | .05 | .94 | 1.06 |  |  |
|  | Age | -5.08 | 1.81 | -.12 | -2.80 | .005 | -.11 | -.13 | -.12 | .99 | 1.01 |  |  |
|  | Ethnicity | 0.07 | 0.05 | .06 | 1.36 | .176 | .06 | .06 | .06 | .97 | 1.03 |  |  |
|  | Whether they did E-Prime portion | -0.18 | 0.13 | -.06 | -1.34 | .182 | -.06 | -.06 | -.06 | .97 | 1.03 |  |  |
|  | FFMQ | 0.26 | 0.19 | .08 | 1.42 | .157 | .02 | .07 | .06 | .70 | 1.44 |  |  |
|  | Effortful Control | -0.05 | 0.12 | -.02 | -0.43 | .668 | -.04 | -.02 | -.02 | .67 | 1.50 |  |  |
|  | Survey Time Estimation | 0.23 | 0.13 | .08 | 1.78 | .077 | .07 | .08 | .08 | .96 | 1.05 |  |  |
|  | CFC Future | 0.01 | 0.01 | .04 | 0.94 | .347 | .03 | .04 | .04 | .93 | 1.07 |  |  |
|  | CFC Immediate | 0.05 | 0.01 | .23 | 5.02 | <.001 | .23 | .23 | .22 | .91 | 1.10 |  |  |
| *Note.* FFMQ = Five Facet Mindfulness Questionnaire, ATQ = Adult Temperament Questionnaire, SST = Stop Signal Task, TPT = Time Production Task, CFC = Considerations of Future Consequences Scale, VIF = Variance Inflation Factors. β = standardized betas. *p* < .001. * *p* < .05. *** *p* < .001. Step 1: *R^2^* = .02, Adjusted *R^2^* = .01; Step 2: *R^2^* = .02, Adjusted *R^2^* = .00, *R^2^* Δ *=* .00, *p* = .743; Step 3: *R^2^* = .06, Adjusted *R^2^* = .04, *R^2^* Δ *=* .04, *p* < .001. | | | | | | | | | | | | | |

**Table 18.** *Summary of Blockwise Multiple Regression Analysis of Survey Variables on Total Sample--Subscales (N = 474)*

|  | | *B* | *SE* | β | *t* | *p* | Zero-Order | Partial | Part | VIF |
| --- | --- | --- | --- | --- | --- | --- | --- | --- | --- | --- |
| 1 | (Constant) | 4.80 | .77 |  | 6.23 | <.001 |  |  |  |  |
|  | Screen Time App | -0.07 | .13 | -.02 | -0.51 | .61 | -.02 | -.03 | -.02 | 1.02 |
|  | Gender | 0.16 | .12 | .06 | 1.31 | .19 | .06 | .06 | .05 | 1.02 |
|  | Age | -0.08 | .04 | -.100^*^ | -2.19 | .03 | -.11 | -.12 | -.12 | 1.00 |
|  | Ethnicity | -0.03 | .04 | -.04 | -0.76 | .45 | .06 | .07 | .07 | 1.01 |
|  | Whether they did E-Prime portion | -0.17 | .13 | -.06 | -1.25 | .21 | -.06 | -.06 | -.06 | 1.01 |
| 2 | (Constant) | 4.86 | 1.01 |  | 4.81 | <.001 |  |  |  |  |
|  | Screen Time App | -0.09 | .13 | -.03 | -0.65 | .51 | -.02 | -.03 | -.03 | 1.03 |
|  | Gender | 0.20 | .13 | .08 | 1.59 | .11 | .06 | .07 | .07 | 1.11 |
|  | Age | -0.08 | .04 | -.09 | -2.05 | .04 | -.11 | -.11 | -.11 | 1.04 |
|  | Ethnicity | -0.02 | .04 | -.03 | -0.55 | .58 | .06 | .06 | .06 | 1.03 |
|  | Whether they did E-Prime portion | -0.12 | .13 | -.04 | -0.94 | .35 | -.06 | -.05 | -.05 | 1.02 |
|  | FFMQ Observing | 0.01 | .02 | .02 | 0.35 | .73 | .11 | .03 | .03 | 1.45 |
|  | FFMQ Describing | 0.00 | .01 | -.01 | -0.20 | .84 | .01 | .01 | .01 | 1.49 |
|  | FFMQ Act Aware | 0.00 | .02 | -.01 | -0.07 | .94 | -.03 | .02 | .02 | 2.17 |
|  | FFMQ Nonjudge | -0.03 | .01 | -.12 | -2.07 | .04 | -.09 | -.10 | -.10 | 1.58 |
|  | FFMQ Nonreact | 0.03 | .02 | .09 | 1.64 | .10 | .10 | .09 | .08 | 1.48 |
|  | ATQ EC Activation | 0.00 | .01 | .00 | 0.01 | .99 | .01 | .01 | .01 | 1.37 |
|  | ATQ EC Attentional | 0.03 | .02 | .12 | 1.92 | .06 | .04 | .07 | .07 | 1.81 |
|  | ATQ EC Inhibitory | -0.03 | .01 | -.13 | -2.62 | .01 | -.12 | -.14 | -.14 | 1.26 |
| 3 | (Constant) | 3.28 | 1.10 |  | 3.00 | .00 |  |  |  |  |
|  | Screen Time App | -0.06 | .13 | -.02 | -0.49 | .62 | -.02 | -.02 | -.02 | 1.04 |
|  | Gender | 0.17 | .13 | .06 | 1.34 | .18 | .06 | .06 | .06 | 1.13 |
|  | Age | -0.09 | .04 | -.106^*^ | -2.33 | .02 | -.11 | -.12 | -.12 | 1.05 |
|  | Ethnicity | -0.01 | .04 | -.02 | -0.32 | .75 | .06 | .05 | .05 | 1.04 |
|  | Whether they did E-Prime portion | -0.15 | .13 | -.05 | -1.10 | .27 | -.06 | -.06 | -.06 | 1.05 |
|  | FFMQ Observing | 0.01 | .02 | .04 | 0.64 | .52 | .11 | .04 | .04 | 1.54 |
|  | FFMQ Describing | 0.00 | .01 | .00 | 0.08 | .94 | .01 | .03 | .02 | 1.52 |
|  | FFMQ Act Aware | 0.02 | .02 | .07 | 1.07 | .29 | -.03 | .07 | .07 | 2.36 |
|  | FFMQ Nonjudge | -0.02 | .01 | -.112^*^ | -1.98 | .05 | -.09 | -.10 | -.09 | 1.62 |
|  | FFMQ Nonreact | 0.02 | .02 | .06 | 1.12 | .26 | .10 | .06 | .05 | 1.51 |
|  | ATQ EC Activation | 0.01 | .01 | .02 | 0.43 | .67 | .01 | .03 | .03 | 1.46 |
|  | ATQ EC Attentional | 0.02 | .02 | .08 | 1.36 | .17 | .04 | .04 | .04 | 1.85 |
|  | ATQ EC Inhibitory | -0.03 | .01 | -.118^*^ | -2.34 | .02 | -.12 | -.13 | -.12 | 1.28 |
|  | Survey Time Estimation-Mid | 0.16 | .17 | .07 | 0.96 | .34 | .08 | .05 | .05 | 2.26 |
|  | Survey Time Estimation-End | -0.11 | .20 | -.04 | -0.53 | .60 | .06 | .00 | .00 | 2.25 |
|  | CFC Future | 0.00 | .01 | .00 | 0.02 | .98 | .03 | .01 | .01 | 1.29 |
|  | CFC Immediate | 0.04 | .01 | .201^***^ | 4.02 | <.001 | .23 | .21 | .20 | 1.26 |

*Note.* Dependent Variable: Media Multitasking Score.

FFMQ = Five Facet Mindfulness Questionnaire, ATQ EC = Adult Temperament Questionnaire Effortful Control subscale, CFC = Considerations of Future Consequences Scale.

*B* = unstandardized betas; β = standardized betas.

Step 1: *R^2^* = .02, Adjusted *R^2^* = .01

Step 2: *R^2^* = .06, Adjusted *R^2^* = .03, *R^2^* Δ *=* .04, *p* = .018

Step 3: *R^2^* = .09, Adjusted *R^2^* = .06, *R^2^* Δ *=* .04, *p* = .002

* *p* < .05. ** *p* < .01. *** *p* < .001.

## Additional Tables—Study 2

| 16 |  |  |  |  |  |  |  |  |  |  |  |  |  |  |  | 1 |
| --- | --- | --- | --- | --- | --- | --- | --- | --- | --- | --- | --- | --- | --- | --- | --- | --- |
| 15 |  |  |  |  |  |  |  |  |  |  |  |  |  |  | 1 | .30^***^ |
| 14 |  |  |  |  |  |  |  |  |  |  |  |  |  | 1 | -.40^***^ | .01 |
| 13 |  |  |  |  |  |  |  |  |  |  |  |  | 1 | -.09 | -.14^**^ | -.04 |
| 12 |  |  |  |  |  |  |  |  |  |  |  | 1 | .55^***^ | -.09 | -.09 | -.01 |
| 11 |  |  |  |  |  |  |  |  |  |  | 1 | .52^**^ | .37^***^ | -.05 | -.06 | 0.01 |
| 10 |  |  |  |  |  |  |  |  |  | 1 | .80^***^ | .82^***^ | .80^***^ | -.10 | -.12^*^ | -.02 |
| 9 |  |  |  |  |  |  |  |  | 1 | .42^***^ | .23^***^ | .40^***^ | .41^***^ | -.02 | .02 | -.01 |
| 8 |  |  |  |  |  |  |  | 1 | .10^*^ | .24^***^ | .20^***^ | .26^***^ | .14^**^ | .08 | .11^*^ | .08 |
| 7  **Table 19.** *Summary of Zero-Order Correlation Analysis on Cognitive Control and Media Multitasking Variables in Study 2 (N = 381)* |  |  |  |  |  |  | 1 | .35^***^ | -.33^***^ | .63^***^ | .50^***^ | .61^***^ | .45^***^ | .01 | -.03 | -.02  *Note.* MMI = Media Multitasking Index, MM = Real-time in-study Media Multitasking during survey and E-Prime tasks, FFMQ = Five Facet Mindfulness Questionnaire, ATQ = Adult Temperament Questionnaire, EC = Effortful Control, SST = Stop Signal Task, RT = Reaction Time (ms). Omission errors were indicated by misses on “go” arrows and commission errors were indicated by key presses on “stop” arrows. Two-tailed: * *p* < .05. ** *p* < .01. ^***^ *p* < .001 |
| 6 |  |  |  |  |  | 1 | .30^***^ | .27^***^ | .31^***^ | .25^***^ | .21^***^ | .28^***^ | .12^*^ | .05 | .03 | .01 |
| 5 |  |  |  |  | 1 | .121^*^ | .01 | -.20^***^ | .26^***^ | .00 | .01 | .01 | -.01 | .01 | .04 | .03 |
| 4 |  |  |  | 1 | .35^***^ | .69^***^ | .69^***^ | .60^***^ | .60^***^ | .52^***^ | .40^***^ | .53^***^ | .36^***^ | .05 | .07 | -.04 |
| 3 |  |  | 1 | -.03 | .09 | -.05 | -.07 | -.03 | -.02 | -.04 | -.05 | -.07 | -.09 | -.05 | .04 | -.01 |
| 2 |  | 1 | .36^***^ | -.09 | .02 | -.07 | -.19^***^ | -.02 | -.01 | -.10^*^ | -.08 | -.05 | -.09 | -.08 | .00 | -.04 |
| 1 | 1 | .15^**^ | .10^*^ | **-.17^**^** | **.07** | **-.11^*^** | **-.20^***^** | **-.12^*^** | **-.11^*^** | **-.12^*^** | **-.08** | **-.11^*^** | **-.10** | .06 | **.10^*^** | **-.04** |
| Variables | 1. MMI | 2. MMT Survey | 3. MMT E-Prime | 4. FFMQ | 5. FFMQ Observing | 6. FFMQ Describing | 7. FFMQ Act Aware | 8. FFMQ Nonjudge | 9. FFMQ Nonreact | 10. ATQ EC | 11. ATQ EC Activation | 12. ATQ EC Attentional | 13. ATQ EC Inhibitory | 14. SST Go RT | 15. sqrt SST Omission | 16. sqrt SST Commission |

**Table 20.** *Summary of Zero-Order Correlation Analysis on Time Perspective and Media Multitasking Variables in Study 2 (N = 381)*

| Variables | 1 | 2 | 3 | 4 | 5 | 6 | 7 | 8 | 9 |
| --- | --- | --- | --- | --- | --- | --- | --- | --- | --- |
| 1. MMI | 1 |  |  |  |  |  |  |  |  |
| 2. MMT Survey | .15^**^ | 1 |  |  |  |  |  |  |  |
| 3. MMT E-Prime | .10^*^ | .36^***^ | 1 |  |  |  |  |  |  |
| 4. STE (Mid) | .23^***^ | **.06** | .10 | 1 |  |  |  |  |  |
| 5. STE (End) | .13^*^ | **.03** | .02 | .46^***^ | 1 |  |  |  |  |
| 6. log STE (Avg) | .16^***^ | **.05** | .03 | .86^***^ | .88^***^ | 1 |  |  |  |
| 7. CFC Future | **-.01** | -.09 | -.02 | -.08 | -.12^*^ | -.10 | 1 |  |  |
| 8. CFC Immediate | **.14^**^** | .14^**^ | .05 | .10 | .05 | .08 | -.47^***^ | 1 |  |
| 9. MCQ log *k* | **.16^**^** | .07 | .00 | -.05 | .000 | -.01 | -.12^**^ | .15^**^ | 1 |
| *Note*. MMI = Media Multitasking Index, MMT = Media Multitasking, STE = Survey Time Estimation, CFC = Considerations of Future Consequences scale, MCQ = Money Choice Questionnaire.   \| Two-tailed significance: * *p* < .05. ** *p* < .01. *** *p* < .001. \| \| --- \| | | | | | | | | | |

## Exploratory Analyses with Additional Media Multitasking Variables

**Study 1**

Cognitive Control. Exploratory analyses that examined the associations between cognitive control variables and specific activities as foreground or background tasks were also conducted. After controlling for demographics, the use of a screen time app, and in-study multitasking, playing audio in the background was negatively associated with the nonjudging of inner experience facet of mindfulness (*r* = -.134, *p* = .004) and self-reported inhibitory control (*r* = -.130, p = .005). Texting as a background activity was associated with both lower self-reported inhibitory control (*r* = -.135, *p* = .004) and behavioral inhibitory control (i.e., commission errors on Stop Signal Task; *r* = .127, *p* = .032). Additional correlation analyses between cognitive control variables and specific activities, as well as combinations of activities, can be found in Table 14. Due to the large number of tests, an alpha error correction was applied—only results with *p*-values smaller than .005 were considered significant. These correlations are in bold.

**Table 21.** *Summary of Correlation Analyses on Cognitive Control and Specific Media Multitasking Variables as Primary and Secondary Tasks, Along with Specific Task Combinations, Controlling for Age, Gender, Ethnicity, Whether Participants Used Screen Time App and Multitasked During Survey (N = 288 – 477)*

| Variables | FFMQ Act Aware | FFMQ Nonjudge | FFMQ Nonreact | ATQ EC Activation | ATQ EC Inhibitory | SST Go RT | SST Commission |
| --- | --- | --- | --- | --- | --- | --- | --- |
| MMI Games | -.11 | -.11 | .05 | .00 | -.05 | -.13 | -.02 |
| MMI Audio | -.10 | -.13^**^ | .07 | -.01 | -.06 | -.04 | .07 |
| MMI HW | .01 | -.05 | .04 | .10 | -.02 | .03 | .10 |
| Audio in BG | -.10 | -.12^**^ | .10 | .01 | -.12^**^ | -.09 | .10 |
| Texting in BG | -.02 | -.08 | .04 | .05 | -.13^**^ | .00 | .13 |
| Videos in BG | -.07 | -.08 | .08 | -.01 | -.11 | .01 | .06 |
| Face in BG | .02 | -.02 | .11 | -.04 | -.12^**^ | .08 | .05 |
| Phone in BG | -.03 | -.07 | .14^**^ | -.02 | -.12^**^ | -.01 | .06 |
| Social Media in BG | -.02 | -.06 | .03 | .01 | -.13^**^ | -.03 | .10 |
| Face + Games | -.10 | -.09 | .04 | -.04 | -.05 | -.17^**^ | -.05 |
| Games + Audio | -.07 | -.13^**^ | .04 | .08 | -.03 | -.16^**^ | .12 |

*Note.* MMI = Media Multitasking Index, HW = Homework, BG = Background, FFMQ = Five Facet Mindfulness Questionnaire, ATQ = Adult Temperament Questionnaire, EC = Effortful Control, SST = Stop Signal Task, RT = Reaction Time (ms). Commission errors were indicated by key presses on “stop” arrows. Two-tailed: * *p* < .01, ** *p* < .005

Time Perspective. Exploratory analyses that examined the associations between time perspective variables and specific activities as foreground or background tasks were also conducted. After controlling for demographics, the use of a screen time app, and in-study multitasking, media multitasking during video content viewing (*r* = .20, *p* < .001), playing audio in the background (*r* = .19, 95%CI = [.099, .274]), texting as a background activity (*r* = .15, 95%CI = [.064, .240]), and watching videos as a background activity (*r* = .24, 95%CI = [.151, .324]) all correlated significantly with having an immediate-focused time perspective. Frequently watching video content while performing other tasks was associated with overestimating the time it took to complete the survey (*r* = .19, *p* < .001). Interestingly, multitasking while working on homework was associated with the future-focused subscale of the CFC (*r* = .12, *p* = .007), but this was found to mainly be driven by combining homework with print media (*r* = .16, *p* < .001). Additional correlation analyses between time perspective variables and specific activities, as well as combinations of activities, can be found in Table 15. Due to the large number of tests, an alpha error correction was applied—only results with *p*-values smaller than .002 were considered significant. These correlations are in bold.

**Table 22.** *Summary of Correlation Analyses on Time Perspective and Specific Media Multitasking Variables as Primary and Secondary Tasks, Along with Specific Task Combinations, Controlling for Age, Gender, Ethnicity, Whether Participants Used Screen Time App and Multitasked During Survey (N = 288 – 477)*

| Variables | Survey Time Estimation (Average) | Time Production Task (Average) | CFC Future | CFC Immediate | |
| --- | --- | --- | --- | --- | --- |
| MMI Games | .06 | .03 | -.01 | .14^*^ | |
| MMI Social | .04 | -.02 | .03 | .09 | |
| MMI Non-Social | .10 | -.07 | -.03 | .12^*^ | |
| MMI Audio | .04 | -.04 | .02 | .14^*^ | |
| MMI Phone | .02 | .05 | .01 | .14^*^ | |
| MMI Texting | .02 | -.06 | -.04 | .08 | |
| MMI Video | .04 | .02 | .00 | .20^**^ | |
| MMI HW | .01 | -.04 | .12^*^ | .07 | |
| MMI Face-to-Face | -.10 | -.10 | -.04 | .02 | |
| Audio in BG | .05 | -.09 | -.01 | .19^**^ | |
| Texting in BG | .01 | -.11 | .07 | .15^**^ | |
| Videos in BG | .12 | .00 | .01 | .22^**^ | |
| Face in BG | .06 | -.11 | -.01 | .16^**^ | |
| Phone in BG | .02 | -.05 | .01 | .22^**^ | |
| Social Media in BG | .03 | -.12 | .04 | .19^**^ | |
| Face + Games | .17^**^ | -.01 | -.02 | .23^**^ | |
| Audio + Face | .03 | -.16^*^ | -.01 | .11 | |
| Audio + Video | .123^*^ | .06 | -.07 | .20^**^ | |
| NonSocial + Face | .07 | -.07 | -.03 | .129^*^ | |
| NonSocial + Games | .18^**^ | .05 | -.09 | .25^**^ | |
| Social + Games | .15^*^ | .05 | -.06 | .22^**^ | |
| Games + Social | .05 | -.05 | -.06 | .21^**^ | |
| Games + NonSocial | .12 | -.03 | .01 | .21^**^ | |
| Games + Audio | -.03 | .04 | .02 | .11 | |
| Video + Audio | .12^*^ | .01 | -.05 | .21^**^ | |
| HW + Face | .01 | -.03 | .04 | .14^*^ | |
| HW + Print | .05 | .07 | .16^**^ | .03 | |
| *Note*. MMI = Media Multitasking Index, HW = Homework, BG = Background, CFC = Considerations of Future Consequences scale. Two-tailed significance: * *p* < .01 ** *p* < .002. | | | | |  |

**Study 2**

Cognitive Control. MMI Social Media was a leading factor in the correlations between MMI and lower cog control, apparently. (all controlling for demos, screentime app, and in-study mmt): *r* = -.14, p *= .*008 with overall FFMQ, -.13, *p* = .012 with acting with awareness, -.17, *p* = .001 with overall EC and -.18, *p* = .001 with attentional control and -.13, *p* = .011 with inhibitory control. Similar to social media, for video and games. MMI HW no sig associations, interesting. Some for texting in the BG. Social media in BG is *r* = -0.18, *p* = .001 with FFMQ AA. Audio in BG has some with FFMQs, but not others.

Time Perspective. (All controlling for demos, screen app, and in-study multitasking): MMI Texting was also associated with log k (*r* = .16, *p* = .004). MMI Social with log k *r* = .13, *p* = .015. MMI Video also immediate (*r* = .13, *p* = .015) and log k (*r* = .24, *p* < .001). Games with immediate (*r* = .18, *p* = .001). Homework with future (*r* = .15, *p* = .008), like in Study 1. Texting in the BG: log *k* = .19, *p* < .001. Social in BG: log *k* *r* = .16, *p* = .003. Phone in BG: log *k r* = .17, *p* = .002. audio in BG log *k r* = .16, *p* = .003. Video in BG log *k r =* .16, *p* = .004. Face in BG log *k r* = .16, *p* = .003

## Sensitivity Analyses for All Hypotheses

**Study 1**

Hypothesis 1: Effortful control will be negatively associated with media multitasking.

- Test family: Exact: Correlation: Bivariate normal model. One-tailed, alpha error prob = .05, power = .8, total sample size = 486, H0 = 0. Can find effect as small as *r* = .11.

Hypothesis 2: Inhibitory control will be negatively associated with media multitasking.

- a: self-reported
  - Test family: Exact: Correlation: Bivariate normal model. One-tailed, alpha error prob = .05, power = .8, total sample size = 486, H0 = 0. Can find effect as small as *r* = .11.
- b: objective (commission and omission errors on Stop Signal Task)
  - Test family: Exact: Correlation: Bivariate normal model. One-tailed, alpha error prob = .05, power = .8, total sample size = 300, H0 = 0. Can find effect as small as *r* = .14.

Hypothesis 3: Mindfulness will be negatively associated with media multitasking.

- Test family: Exact: Correlation: Bivariate normal model. One-tailed, alpha error prob = .05, power = .8, total sample size = 486, H0 = 0. Can find effect as small as *r* = .11.

Hypothesis 4: Having an immediate-focused time perspective will be associated with more media multitasking.

- Test family: Exact: Correlation: Bivariate normal model. One-tailed, alpha error prob = .05, power = .8, total sample size = 486, H0 = 0. Can find effect as small as *r* = .11.

Hypothesis 5: Real-time media multitasking will be associated with time passage underestimation.

- Test family: Exact: Correlation: Bivariate normal model. One-tailed, alpha error prob = .05, power = .8, total sample size = 483, H0 = 0. Can find effect as small as *r* = .11.

**Study 2**

Hypothesis 1: Effortful control will be negatively associated with media multitasking.

- Test family: Exact: Correlation: Bivariate normal model. One-tailed, alpha error prob = .05, power = .8, total sample size = 381, H0 = 0. Can find effect as small as *r* = .13.

Hypothesis 2: Inhibitory control will be negatively associated with media multitasking.

- a: self-reported
  - Test family: Exact: Correlation: Bivariate normal model. One-tailed, alpha error prob = .05, power = .8, total sample size = 381, H0 = 0. Can find effect as small as *r* = .13.
- b: objective (commission and omission errors on Stop Signal Task)
  - Test family: Exact: Correlation: Bivariate normal model. One-tailed, alpha error prob = .05, power = .8, total sample size = 376, H0 = 0. Can find effect as small as *r* = .13.

Hypothesis 3: Mindfulness will be negatively associated with media multitasking.

- Test family: Exact: Correlation: Bivariate normal model. One-tailed, alpha error prob = .05, power = .8, total sample size = 381, H0 = 0. Can find effect as small as *r* = .13.

Hypothesis 4: Having an immediate-focused time perspective will be associated with more media multitasking.

- Test family: Exact: Correlation: Bivariate normal model. One-tailed, alpha error prob = .05, power = .8, total sample size = 381, H0 = 0. Can find effect as small as *r* = .13.

Hypothesis 5: Real-time media multitasking will be associated with time passage underestimation.

- Test family: Exact: Correlation: Bivariate normal model. One-tailed, alpha error prob = .05, power = .8, total sample size = 381, H0 = 0. Can find effect as small as *r* = .13.

Hypothesis 6: Steeper delay discounting (preference for short-term reward) will be associated with more media multitasking.

- Test family: Exact: Correlation: Bivariate normal model. One-tailed, alpha error prob = .05, power = .8, total sample size = 379, H0 = 0. Can find effect as small as *r* = .13.

## Structural Regression Modeling

We conducted structural regression modeling using a combined sample (*N* = 868) using the R Lavaan package. The following criteria were used to determine model fit during the structural equation modeling portion of analysis. An ideal fit comprised a non-significant χ^2^; a comparative fit index (CFI) value greater than 0.96; and root mean square of approximation (RMSEA) and standardized root mean squared residuals (SRMR) values below 0.06 (Byrne, 2013; Hair et al., 1998). It is important to note that the χ^2^ statistic is easily skewed by sample size, so it is important to present it alongside the other fit indices. An acceptable but not ideal level of fit for the CFI is 0.90, and for the RMSEA and SRMR is below 0.10. The Modification Indices function was used to improve model fit, with the stipulation that only correlated errors would be added, the correlations would be added one by one, and there would be no cross-loadings between latent variables.

The result of the structural regression modeling can be found in Figure 2. The model had an adequate fit (CFI = .75 [scaled] or .88 [standard], RMSEA = .03 [scaled] or .02 [standard], SRMR = .06 [both scaled and standard], χ^2^(194) = 242.71, *p* = .010 [scaled] or χ^2^ (194) = 227.04, *p* = .05 [standard]). The strongest predictor of media multitasking frequency were the CFC immediate subscale (β = .35), survey overestimation (β = .20) and self-reported inhibitory control (β = -.18). We also created a model that included gender and ethnicity, but the loadings did not change significantly from the original.

**Figure 1.** *Structural Regression Model using Combined Dataset (N = 868).*


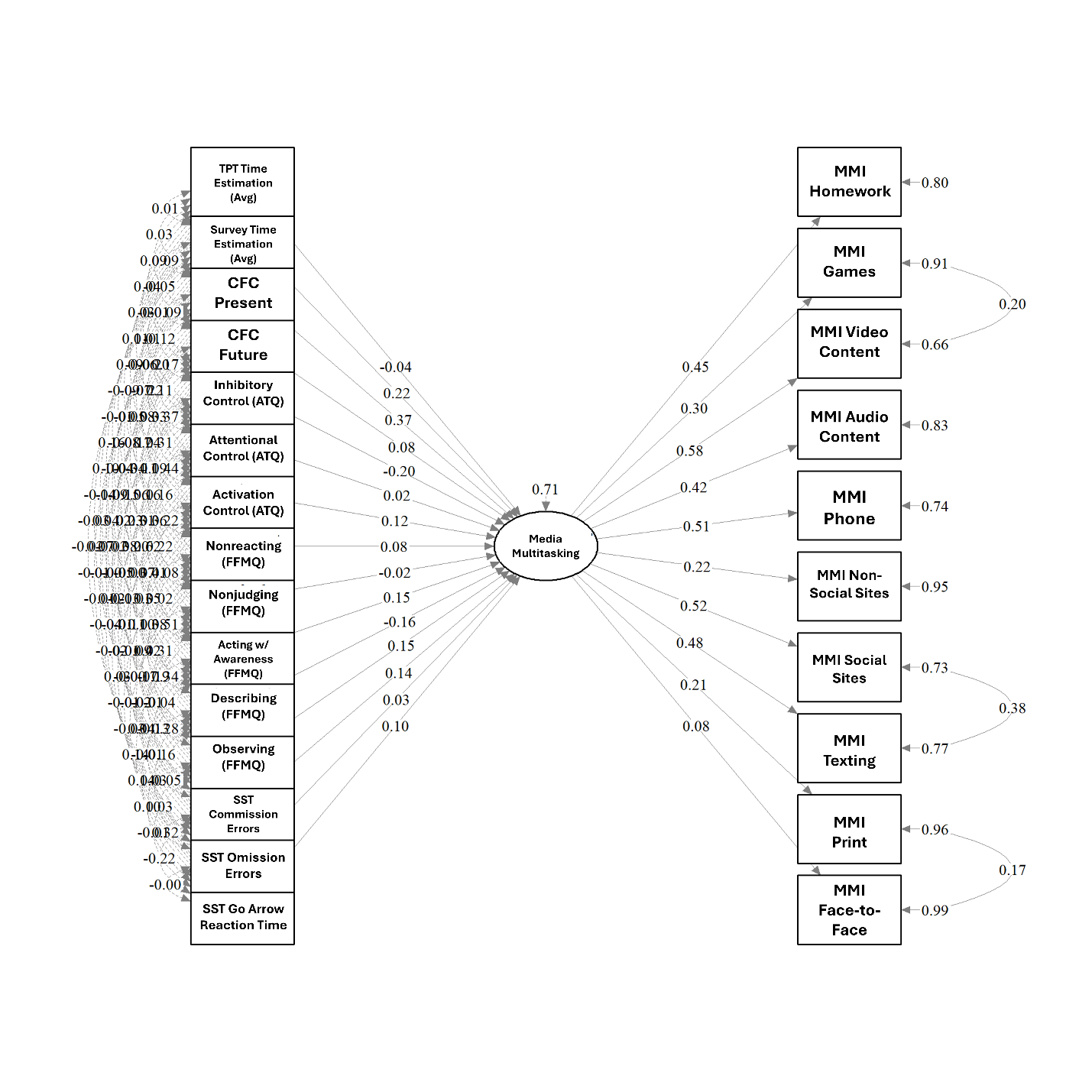


*Note*. TPT = Time Production Task, CFC = Considerations of Future Consequences Scale. ATQ = Adult Temperament Questionnaire, FFMQ = Five Factor Mindfulness Questionnaire. SST = Stop Signal Task. MMI = Media Multitasking Index. Standardized paths are shown.

## Analyses with ADHD Diagnosis

**Study 1**

**Table 23.** *Blockwise Regression Predicting Media Multitasking Frequency (MMI), with ADHD as Predictor, Study 1*

| Model | | Unstandardized Coefficients | | Standardized Coefficients | *t* | *p* | Collinearity Statistics | |
| --- | --- | --- | --- | --- | --- | --- | --- | --- |
|  |  | *B* | *SE* | β |  |  | Tolerance | VIF |
| 1 | (Constant) | 6.29 | 3.00 |  | 2.10 | .04 |  |  |
|  | ScreenApp | -0.08 | 0.17 | -.03 | -0.49 | .62 | 0.98 | 1.02 |
|  | Age | -2.76 | 2.34 | -.07 | -1.18 | .24 | 1.00 | 1.00 |
|  | Gender | 0.22 | 0.15 | .08 | 1.43 | .15 | 0.97 | 1.03 |
|  | Ethnicity | 0.05 | 0.06 | .04 | 0.75 | .45 | 1.00 | 1.00 |
|  | ADHD | 0.00 | 0.00 | -.13 | -2.16 | .03 | 0.99 | 1.01 |
| 2 | (Constant) | 6.49 | 3.29 |  | 1.97 | .05 |  |  |
|  | ScreenApp | -0.09 | 0.17 | -.03 | -0.53 | .60 | 0.97 | 1.03 |
|  | Age | -2.94 | 2.35 | -.07 | -1.25 | .21 | 0.99 | 1.01 |
|  | Gender | 0.18 | 0.16 | .07 | 1.08 | .28 | 0.87 | 1.15 |
|  | Ethnicity | 0.06 | 0.06 | .06 | 1.02 | .31 | 0.98 | 1.02 |
|  | ADHD | 0.00 | 0.00 | -.13 | -2.13 | .03 | 0.98 | 1.02 |
|  | FFMQ | 0.17 | 0.24 | .05 | 0.72 | .47 | 0.68 | 1.47 |
|  | Effortful Control | -0.12 | 0.15 | -.06 | -0.78 | .43 | 0.68 | 1.47 |
|  | SST Go RT | 0.00 | 0.00 | -.02 | -0.32 | .75 | 0.84 | 1.19 |
|  | SST Omiss | 0.10 | 0.09 | .07 | 1.07 | .29 | 0.85 | 1.17 |
|  | SST Comiss | 0.12 | 0.11 | .07 | 1.08 | .28 | 0.81 | 1.24 |
| 3 | (Constant) | 4.05 | 3.23 |  | 1.25 | .21 |  |  |
|  | ScreenApp | -0.06 | 0.16 | -.02 | -0.35 | .72 | 0.97 | 1.03 |
|  | Age | -3.08 | 2.25 | -.08 | -1.37 | .17 | 0.99 | 1.01 |
|  | Gender | 0.17 | 0.16 | .07 | 1.09 | .28 | 0.85 | 1.17 |
|  | Ethnicity | 0.05 | 0.06 | .05 | 0.85 | .39 | 0.93 | 1.07 |
|  | ADHD | 0.00 | 0.00 | -.12 | -2.16 | .03 | 0.97 | 1.03 |
|  | FFMQ | 0.30 | 0.23 | .09 | 1.33 | .18 | 0.66 | 1.51 |
|  | Effortful Control | -0.06 | 0.15 | -.03 | -0.42 | .67 | 0.64 | 1.55 |
|  | SST Go RT | 0.00 | 0.00 | -.02 | -0.27 | .79 | 0.84 | 1.20 |
|  | SST Omiss | 0.10 | 0.09 | .07 | 1.08 | .28 | 0.85 | 1.18 |
|  | SST Comiss | 0.13 | 0.11 | .08 | 1.25 | .21 | 0.79 | 1.27 |
|  | Survey Est (Avg) | 0.45 | 0.17 | .15 | 2.70 | .01 | 0.95 | 1.05 |
|  | TPT Est (Avg) | -0.49 | 0.37 | -.08 | -1.35 | .18 | 0.92 | 1.09 |
|  | CFC Future | 0.02 | 0.01 | .09 | 1.51 | .13 | 0.90 | 1.11 |
|  | CFC Immediate | 0.05 | 0.01 | .25 | 4.34 | .00 | 0.91 | 1.10 |
| *Note.* Dependent Variable: Media Multitasking Score.  FFMQ = Five Facet Mindfulness Questionnaire, SST = Stop Signal Task, TPT = Time Production Task, CFC = Considerations of Future Consequences Scale.  *B* = unstandardized betas; β = standardized betas.  Step 1: *R^2^* = .03, Adjusted *R^2^* = .01  Step 2: *R^2^* = .04, Adjusted *R^2^* = .01, *R^2^* Δ *=* .02, *p* = .499  Step 3: *R^2^* = .14, Adjusted *R^2^* = .10, *R^2^* Δ *=* .10, *p* < .001. F(14, 278) = 3.25, *p* < .001  * *p* < .05. ** *p* < .01. *** *p* < .001 | | | | | | | | |

**Study 2**

**Table 24.** *Blockwise Regression Predicting Media Multitasking Frequency (MMI), with ADHD as Predictor, Study 2*

| Model | | Unstandardized Coefficients | | Standardized Coefficients | *t* | *p* | Collinearity Statistics | |
| --- | --- | --- | --- | --- | --- | --- | --- | --- |
|  |  | *B* | *SE* | β |  |  | Tolerance | VIF |
| 1 | (Constant) | 7.81 | 3.12 |  | 2.50 | .01 |  |  |
|  | ScreenApp | 0.12 | 0.14 | .05 | 0.90 | .37 | 0.99 | 1.01 |
|  | Age | 0.17 | 0.12 | .07 | 1.41 | .16 | 0.94 | 1.07 |
|  | Gender | -4.34 | 2.43 | -.09 | -1.78 | .08 | 0.96 | 1.04 |
|  | Ethnicity | -0.02 | 0.06 | -.01 | -0.29 | .77 | 0.99 | 1.01 |
|  | Financial Stress | 0.01 | 0.00 | .17 | 3.32 | .00 | 0.97 | 1.03 |
|  | ADHD | 0.07 | 0.08 | .04 | 0.82 | .42 | 0.99 | 1.01 |
| 2 | (Constant) | 9.02 | 3.32 |  | 2.71 | .01 |  |  |
|  | ScreenApp | 0.16 | 0.14 | .06 | 1.18 | .24 | 0.98 | 1.02 |
|  | Age | 0.15 | 0.12 | .07 | 1.24 | .22 | 0.88 | 1.14 |
|  | Gender | -4.12 | 2.44 | -.09 | -1.69 | .09 | 0.95 | 1.06 |
|  | Ethnicity | -0.03 | 0.06 | -.03 | -0.59 | .55 | 0.95 | 1.05 |
|  | Financial Stress | 0.01 | 0.00 | .14 | 2.71 | .01 | 0.90 | 1.11 |
|  | ADHD | 0.05 | 0.09 | .03 | 0.53 | .60 | 0.88 | 1.13 |
|  | FFMQ | -0.01 | 0.00 | -.12 | -2.01 | .04 | 0.68 | 1.47 |
|  | Effortful Control | 0.00 | 0.01 | .01 | 0.19 | .85 | 0.58 | 1.72 |
|  | SST Go RT | 0.00 | 0.00 | -.02 | -0.31 | .76 | 0.79 | 1.27 |
|  | SST Omiss | 0.22 | 0.10 | .14 | 2.29 | .02 | 0.71 | 1.40 |
|  | SST Comiss | -0.12 | 0.09 | -.07 | -1.37 | .17 | 0.87 | 1.15 |
| 3 | (Constant) | 6.55 | 3.43 |  | 1.91 | .06 |  |  |
|  | ScreenApp | 0.18 | 0.14 | .07 | 1.36 | .18 | 0.97 | 1.03 |
|  | Age | 0.16 | 0.12 | .07 | 1.31 | .19 | 0.87 | 1.14 |
|  | Gender | -3.15 | 2.45 | -.07 | -1.29 | .20 | 0.92 | 1.09 |
|  | Ethnicity | -0.04 | 0.06 | -.03 | -0.61 | .54 | 0.95 | 1.05 |
|  | Financial Stress | 0.01 | 0.00 | .13 | 2.37 | .02 | 0.89 | 1.12 |
|  | ADHD | 0.02 | 0.09 | .01 | 0.26 | .79 | 0.87 | 1.15 |
|  | FFMQ | -0.01 | 0.00 | -.10 | -1.57 | .12 | 0.65 | 1.54 |
|  | Effortful Control | 0.00 | 0.01 | .00 | -0.03 | .97 | 0.52 | 1.92 |
|  | SST Go RT | 0.00 | 0.00 | -.02 | -0.37 | .71 | 0.78 | 1.28 |
|  | SST Omiss | 0.19 | 0.09 | .12 | 2.04 | .04 | 0.70 | 1.42 |
|  | SST Comiss | -0.11 | 0.09 | -.07 | -1.22 | .22 | 0.86 | 1.16 |
|  | Survey Est (Avg) | 1.26 | 0.54 | .12 | 2.35 | .02 | 0.93 | 1.07 |
|  | CFC Future | 0.02 | 0.01 | .12 | 2.00 | .05 | 0.72 | 1.39 |
|  | CFC Immediate | 0.02 | 0.01 | .12 | 1.96 | .05 | 0.64 | 1.56 |
| 4 | (Constant) | 8.01 | 3.43 |  | 2.34 | .02 |  |  |
|  | ScreenApp | 0.19 | 0.13 | .07 | 1.45 | .15 | 0.97 | 1.03 |
|  | Age | 0.19 | 0.12 | .08 | 1.57 | .12 | 0.87 | 1.15 |
|  | Gender | -3.91 | 2.44 | -.08 | -1.60 | .11 | 0.91 | 1.10 |
|  | Ethnicity | -0.03 | 0.06 | -.03 | -0.55 | .58 | 0.95 | 1.05 |
|  | Financial Stress | 0.00 | 0.00 | .09 | 1.78 | .08 | 0.86 | 1.16 |
|  | ADHD | 0.03 | 0.09 | .02 | 0.29 | .77 | 0.87 | 1.15 |
|  | FFMQ | -0.01 | 0.00 | -.11 | -1.72 | .09 | 0.65 | 1.54 |
|  | Effortful Control | 0.00 | 0.01 | .00 | 0.03 | .98 | 0.52 | 1.92 |
|  | SST Go RT | 0.00 | 0.00 | -.01 | -0.13 | .90 | 0.78 | 1.29 |
|  | SST Omiss | 0.19 | 0.09 | .12 | 2.07 | .04 | 0.70 | 1.42 |
|  | SST Comiss | -0.11 | 0.09 | -.07 | -1.23 | .22 | 0.86 | 1.16 |
|  | Survey Est (Avg) | 1.27 | 0.53 | .12 | 2.40 | .02 | 0.93 | 1.07 |
|  | CFC Future | 0.02 | 0.01 | .13 | 2.21 | .03 | 0.72 | 1.40 |
|  | CFC Immediate | 0.02 | 0.01 | .10 | 1.69 | .09 | 0.63 | 1.58 |
|  | MCQ log K | 0.31 | 0.10 | .16 | 3.01 | .00 | 0.91 | 1.10 |
| *Note.* Dependent Variable: Media Multitasking Score.  FFMQ = Five Facet Mindfulness Questionnaire, SST = Stop Signal Task, TPT = Time Production Task, CFC = Considerations of Future Consequences Scale, MCQ = Money Choice Questionnaire.  *B* = unstandardized betas; β = standardized betas.  Step 1: *R^2^* = .06, Adjusted *R^2^* = .04  Step 2: *R^2^* = .08, Adjusted *R^2^* = .05, *R^2^* Δ *=* .03, *p* = .06  Step 3: *R^2^* = .11, Adjusted *R^2^* = .07, *R^2^* Δ *=* .03, *p* = .01  Step 3: *R^2^* = .13, Adjusted *R^2^* = .10, *R^2^* Δ *=* .02, *p* = .00. F(15, 372) = 3.60, *p* < .001  * *p* < .05. ** *p* < .01. *** *p* < .001 | | | | | | | | |

## Moderation Analyses

We also conducted moderation analyses using the SPSS PROCESS Macro (Hayes, 2012) with the combined sample (*N* = 868). We investigated the moderating effects of cognitive control on the relation between time perspective and outcomes (i.e., media multitasking frequency), was well as time perspective and time estimation.

Both the acting with awareness (interaction = 0.002, *p* = .03, *SE =* .00; R^2^ Δ = .01) facet of mindfulness and the attentional control (interaction = 0.002, *p* = .05, *SE* = .00; R^2^ Δ = .00) subscale of effortful control moderated the relation between present time perspective and media multitasking. Those who had higher acting with awareness and attentional control had a stronger association between short-term goals and media multitasking frequency, which was counter to prior research. However, this is the first time conducting such an analysis with media multitasking as the outcome variable. The association was mostly driven by multitasking while speaking face-to-face with another person and texting.

No aspect of self-control significantly moderated the relation between time perspective and time estimation. However, we did not use a measure of unbalanced time perspective (there are no “optimal” levels of either time perspective using the CFC scale, which are required to calculate imbalance).

**Figure 2.** *Moderation by Acting with Awareness Mindfulness Facet of Relation Between Present Time Perspective and Media Multitasking Frequency*


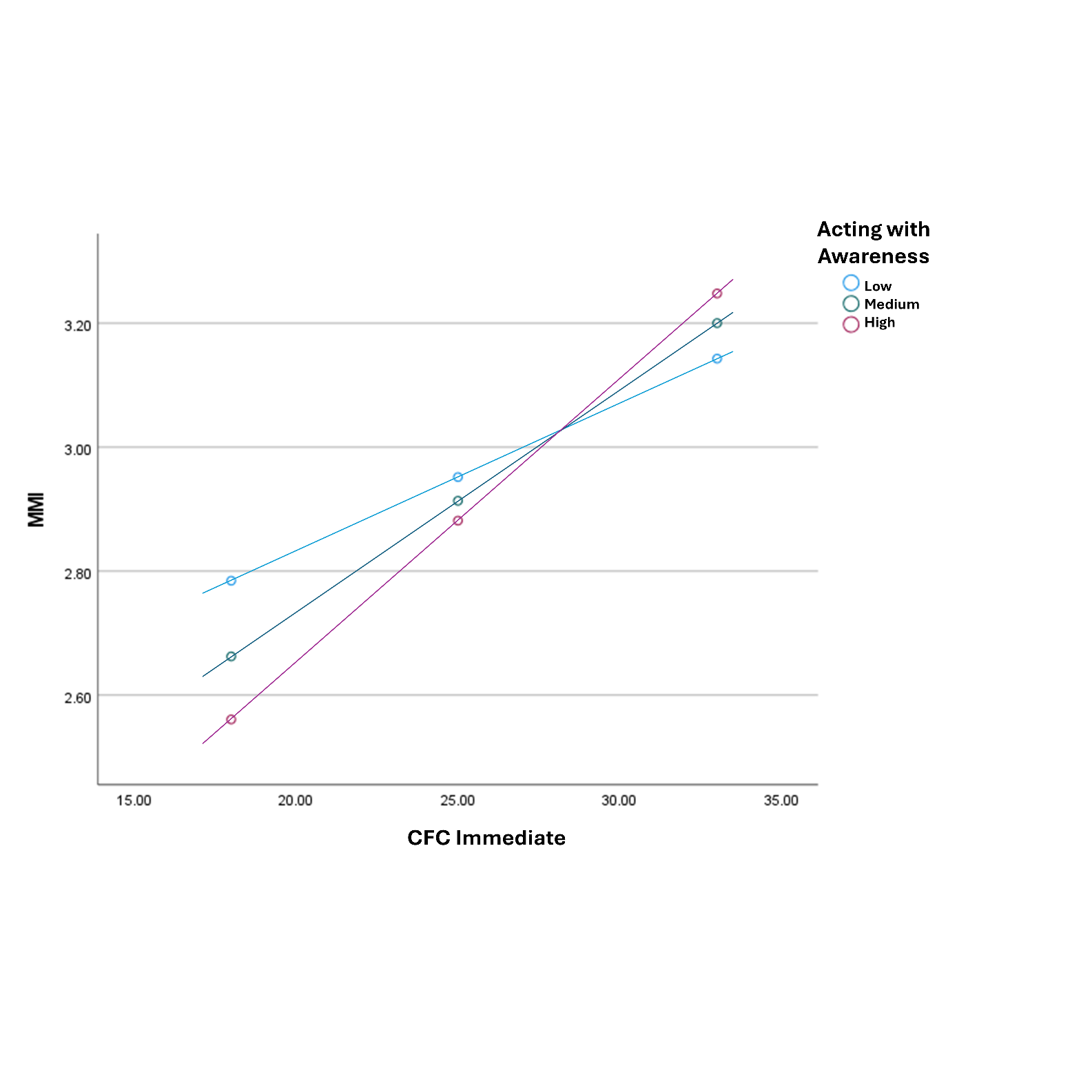


## Regression Analyses with Semi-Partial Correlations

**Study 1**

| **Table 25.** *Summary of Blockwise Multiple Regression Analysis of Survey Variables on Total Sample--Subscales (N = 474)* | | | | | | | | | | | | | |
| --- | --- | --- | --- | --- | --- | --- | --- | --- | --- | --- | --- | --- | --- |
| Model | |  | |  | *t* | *p* | Correlations | | | Collinearity Statistics | | |  |
|  |  | b | *SE* | β |  |  | Zero-order | Partial | Part | Tolerance | VIF |  |  |
| 1 | (Constant) | 6.21 | 3.02 |  | 2.05 | .041 |  |  |  |  |  |  |  |
|  | Screen Time App | -0.09 | 0.17 | -.03 | -0.53 | .600 | -.02 | -.03 | -.03 | .98 | 1.02 |  |  |
|  | Gender | 0.20 | 0.16 | .08 | 1.30 | .196 | .07 | .08 | .08 | .98 | 1.02 |  |  |
|  | Age | -2.71 | 2.35 | -.07 | -1.15 | .250 | -.07 | -.07 | -.07 | 1.00 | 1.00 |  |  |
|  | Ethnicity | 0.05 | 0.06 | .05 | 0.84 | .401 | .05 | .05 | .05 | 1.00 | 1.00 |  |  |
| 2 | (Constant) | 6.47 | 3.32 |  | 1.95 | .052 |  |  |  |  |  |  |  |
|  | Screen Time App | -0.10 | 0.17 | -.03 | -0.57 | .572 | -.02 | -.03 | -.03 | .97 | 1.03 |  |  |
|  | Gender | 0.16 | 0.17 | .06 | 0.97 | .333 | .07 | .06 | .06 | .87 | 1.15 |  |  |
|  | Age | -2.88 | 2.36 | -.07 | -1.22 | .224 | -.07 | -.07 | -.07 | .99 | 1.01 |  |  |
|  | Ethnicity | 0.07 | 0.06 | .07 | 1.10 | .271 | .05 | .07 | .07 | .98 | 1.02 |  |  |
|  | FFMQ | 0.20 | 0.24 | .06 | 0.85 | .397 | .01 | .05 | .05 | .68 | 1.46 |  |  |
|  | Effortful Control | -0.14 | 0.15 | -.07 | -0.92 | .361 | -.05 | -.05 | -.05 | .68 | 1.47 |  |  |
|  | Go RT | 0.00 | 0.00 | -.03 | -0.42 | .677 | .00 | -.03 | -.02 | .84 | 1.19 |  |  |
|  | Omiss_sqrt | 0.09 | 0.10 | .06 | 0.97 | .334 | .08 | .06 | .06 | .85 | 1.17 |  |  |
|  | Comis_sqrt | 0.12 | 0.11 | .07 | 1.10 | .274 | .09 | .07 | .06 | .81 | 1.24 |  |  |
| 03 | (Constant) | 3.86 | 3.25 |  | 1.19 | .236 |  |  |  |  |  |  |  |
|  | Screen Time App | -0.06 | 0.16 | -.02 | -0.39 | .699 | -.02 | -.02 | -.02 | .97 | 1.03 |  |  |
|  | Gender | 0.16 | 0.16 | .06 | 0.99 | .321 | .07 | .06 | .06 | .85 | 1.17 |  |  |
|  | Age | -3.00 | 2.26 | -.08 | -1.33 | .186 | -.07 | -.08 | -.07 | .99 | 1.01 |  |  |
|  | Ethnicity | 0.06 | 0.06 | .06 | 0.99 | .324 | .05 | .06 | .06 | .94 | 1.07 |  |  |
|  | FFMQ | 0.33 | 0.23 | .10 | 1.45 | .148 | .01 | .09 | .08 | .66 | 1.51 |  |  |
|  | Effortful Control | -0.08 | 0.15 | -.04 | -0.57 | .571 | -.05 | -.03 | -.03 | .65 | 1.55 |  |  |
|  | Go RT | 0.00 | 0.00 | -.02 | -0.36 | .723 | .00 | -.02 | -.02 | .84 | 1.19 |  |  |
|  | Omiss_sqrt | 0.09 | 0.09 | .06 | 0.99 | .324 | .08 | .06 | .06 | .85 | 1.18 |  |  |
|  | Comis_sqrt | 0.14 | 0.11 | .08 | 1.31 | .193 | .09 | .08 | .07 | .79 | 1.27 |  |  |
|  | Survey Estimation | 0.46 | 0.17 | .16 | 2.70 | .007 | .16 | .16 | .15 | .95 | 1.05 |  |  |
|  | TPT Estimation | -0.40 | 0.37 | -.06 | -1.10 | .272 | -.07 | -.07 | -.06 | .93 | 1.07 |  |  |
|  | CFC Future | .020 | .012 | .094 | 1.596 | .112 | .06 | .10 | .09 | .91 | 1.10 |  |  |
|  | CFC Immediate | .050 | .011 | .256 | 4.355 | <.001 | -.02 | -.03 | -.03 | .98 | 1.10 |  |  |
| *Note.* FFMQ = Five Facet Mindfulness Questionnaire, ATQ = Adult Temperament Questionnaire, SST = Stop Signal Task, TPT = Time Production Task, CFC = Considerations of Future Consequences Scale. β = standardized betas. * *p* < .05. *** *p* < .001. Step 1: *R^2^* = .01, Adjusted *R^2^* = -.00; Step 2: *R^2^* = .03, Adjusted *R^2^* = -.00, *R^2^* Δ *=* .02, *p* = .068; Step 3: *R^2^* = .13, Adjusted *R^2^* = .09, *R^2^* Δ *=* .10, *p* < .001 | | | | | | | | | | | | | |

**Study 2**

| **Table 26.** *Summary of Blockwise Multiple Regression Analysis on Media Multitasking Index Score (N = 381)* | | | | | | | | | | | |
| --- | --- | --- | --- | --- | --- | --- | --- | --- | --- | --- | --- |
| Model | |  | |  |  |  | Correlations | | | Collinearity Statistics | |
|  |  | b | *SE* | *t* | β | *p* | Zero-order | Partial | Part | Tolerance | VIF |
| 1 | (Constant) | 7.90 | 3.12 |  | 2.53 | .01 |  |  |  |  |  |
|  | STA | 0.13 | 0.14 | .05 | 0.92 | .36 | .05 | .05 | .05 | .99 | 1.01 |
|  | Gender | 0.17 | 0.12 | .07 | 1.42 | .16 | .12 | .07 | .07 | .94 | 1.07 |
|  | Age | -4.39 | 2.43 | -.09 | -1.81 | .07 | -.10 | -.09 | -.09 | .96 | 1.04 |
|  | Ethnicity | -0.02 | 0.06 | -.02 | -0.31 | .76 | -.02 | -.02 | -.02 | .99 | 1.01 |
|  | Financial Stress | 0.01 | 0.00 | .17 | 3.38 | .00 | .18 | .17 | .17 | .97 | 1.03 |
| 2 | (Constant) | 9.16 | 3.31 |  | 2.77 | .01 |  |  |  |  |  |
|  | STA | 0.16 | 0.14 | .06 | 1.20 | .23 | .05 | .06 | .06 | .98 | 1.02 |
|  | Gender | 0.15 | 0.12 | .07 | 1.22 | .23 | .12 | .06 | .06 | .88 | 1.14 |
|  | Age | -4.17 | 2.43 | -.09 | -1.71 | .09 | -.10 | -.09 | -.09 | .95 | 1.06 |
|  | Ethnicity | -0.04 | 0.06 | -.03 | -0.63 | .53 | -.02 | -.03 | -.03 | .96 | 1.05 |
|  | Financial Stress | 0.01 | 0.00 | .14 | 2.70 | .01 | .18 | .14 | .14 | .90 | 1.11 |
|  | FFMQ | -0.01 | 0.01 | -.12 | -1.99 | .05 | -.16 | -.10 | -.10 | .68 | 1.46 |
|  | Effortful Control | 0.00 | 0.01 | .00 | 0.03 | .98 | -.13 | .00 | .00 | .64 | 1.56 |
|  | SST Go RT | 0.00 | 0.00 | -.02 | -0.32 | .75 | .05 | -.02 | -.02 | .79 | 1.27 |
|  | SST Omission | 0.22 | 0.10 | .14 | 2.31 | .02 | .10 | .12 | .12 | .72 | 1.40 |
|  | SST Commission | -0.12 | 0.09 | -.07 | -1.35 | .18 | -.03 | -.07 | -.07 | .87 | 1.15 |
| 3 | (Constant) | 6.60 | 3.42 |  | 1.93 | .06 |  |  |  |  |  |
|  | STA | 0.19 | 0.14 | .07 | 1.37 | .17 | .05 | .07 | .07 | .97 | 1.03 |
|  | Gender | 0.16 | 0.12 | .07 | 1.30 | .20 | .12 | .07 | .07 | .88 | 1.14 |
|  | Age | -3.17 | 2.45 | -.07 | -1.29 | .20 | -.10 | -.07 | -.06 | .92 | 1.09 |
|  | Ethnicity | -0.04 | 0.06 | -.03 | -0.63 | .53 | -.02 | -.03 | -.03 | .95 | 1.05 |
|  | Financial Stress | 0.01 | 0.00 | .13 | 2.37 | .02 | .18 | .12 | .12 | .89 | 1.12 |
|  | FFMQ | -0.01 | 0.01 | -.10 | -1.55 | .12 | -.16 | -.08 | -.08 | .66 | 1.53 |
|  | Effortful Control | 0.00 | 0.01 | -.01 | -0.11 | .91 | -.13 | -.01 | -.01 | .57 | 1.77 |
|  | SST Go RT | 0.00 | 0.00 | -.02 | -0.37 | .71 | .05 | -.02 | -.02 | .78 | 1.28 |
|  | SST Omission | 0.19 | 0.10 | .12 | 2.04 | .04 | .10 | .11 | .10 | .70 | 1.43 |
|  | SST Commission | -0.11 | 0.09 | -.07 | -1.21 | .23 | -.03 | -.06 | -.06 | .86 | 1.16 |
|  | Survey Time Estimation | 1.27 | 0.53 | .12 | 2.38 | .02 | .16 | .12 | .12 | .94 | 1.07 |
|  | CFC Future | 0.02 | 0.01 | .12 | 2.00 | .05 | .00 | .11 | .10 | .72 | 1.39 |
|  | CFC Immediate | 0.02 | 0.01 | .12 | 1.99 | .05 | .15 | .10 | .10 | .64 | 1.56 |
| 4 | (Constant) | 8.06 | 3.42 |  | 2.36 | .02 |  |  |  |  |  |
|  | STA | 0.20 | 0.13 | .07 | 1.46 | .14 | .05 | .08 | .07 | .97 | 1.03 |
|  | Gender | 0.19 | 0.12 | .08 | 1.56 | .12 | .12 | .08 | .08 | .87 | 1.15 |
|  | Age | -3.92 | 2.43 | -.08 | -1.61 | .11 | -.10 | -.09 | -.08 | .91 | 1.10 |
|  | Ethnicity | -0.03 | 0.06 | -.03 | -0.57 | .57 | -.02 | -.03 | -.03 | .95 | 1.05 |
|  | Financial Stress | 0.00 | 0.00 | .09 | 1.77 | .08 | .18 | .09 | .09 | .86 | 1.16 |
|  | FFMQ | -0.01 | 0.01 | -.10 | -1.71 | .09 | -.16 | -.09 | -.08 | .65 | 1.53 |
|  | Effortful Control | 0.00 | 0.01 | .00 | -0.06 | .96 | -.13 | .00 | .00 | .57 | 1.77 |
|  | SST Go RT | 0.00 | 0.00 | -.01 | -0.13 | .89 | .05 | -.01 | -.01 | .78 | 1.29 |
|  | SST Omission | 0.20 | 0.09 | .12 | 2.08 | .04 | .10 | .11 | .10 | .70 | 1.43 |
|  | SST Commission | -0.11 | 0.09 | -.07 | -1.22 | .22 | -.03 | -.06 | -.06 | .86 | 1.16 |
|  | Survey Time Estimation | 1.28 | 0.53 | .12 | 2.43 | .02 | .16 | .13 | .12 | .94 | 1.07 |
|  | CFC Future | 0.02 | 0.01 | .13 | 2.21 | .03 | .00 | .12 | .11 | .72 | 1.40 |
|  | CFC Immediate | 0.02 | 0.01 | .11 | 1.72 | .09 | .15 | .09 | .09 | .64 | 1.57 |
|  | MCQ *log* K | 0.31 | 0.10 | .16 | 3.01 | .00 | .16 | .16 | .15 | .91 | 1.10 |
| *Note.* STA = Use of Screen Time App, FFMQ = Five Facet Mindfulness Questionnaire, ATQ EC = Adult Temperament Questionnaire Effortful Control, SST = Stop Signal Task, STE = Survey Time Estimation, CFC = Considerations of Future Consequences Scale, MCQ = Money Choice Questionnaire. β = standardized betas. * *p* < .05. ** *p* < .01. *** *p* < .001. Step 1: *R^2^* = .05, Adjusted *R^2^* = .04; Step 2: *R^2^* = .08, Adjusted *R^2^* = .06, *R^2^* Δ *=* .03, *p* = .05; Step 3: *R^2^* = .11, Adjusted *R^2^* = .08, *R^2^* Δ *=* .03, *p* = .011 Step 4: *R^2^* = .131, Adjusted *R^2^* = .10, *R^2^* Δ *=* .02, *p* = .003. | | | | | | | | | | | |
